# Contextual determinants of implementation of stakeholder-selected quality indicators in Indian intensive care units: a mixed-methods evaluation within a national registry network

**DOI:** 10.3389/frhs.2026.1872641

**Published:** 2026-07-27

**Authors:** Vrindha Pari, Bharath Kumar Tirupakuzhi Vijayaraghavan, Swagata Tripathy, Raymond Dominic Savio, Pratheema Ramachandran, Sandeep Shyamsundar, Meghena Mathew, Vanishree Chandran, Rashan Haniffa, Justine Davies, Abi Beane

**Affiliations:** 1 Department of Applied Health Research, University of Birmingham, Birmingham, United Kingdom; 2 Department of Critical Care, Apollo Hospitals, Chennai, India; 3 Centre for Inflammation Research, University of Edinburgh, Edinburgh, Scotland, United Kingdom; 4 Department of Anaesthesia and Critical Care, All India Institute of Medical Sciences (AIIMS), Bhubaneswar, India; 5 Department of Critical Care, Apollo Proton Cancer Center, Chennai, India; 6 Department of Critical Care, Apollo Speciality Hospitals, OMR, Chennai, India; 7 Department of Critical Care, Apollo Speciality Hospitals, Teynampet, Chennai, India; 8 Department of Critical Care, Apollo First Med Hospitals, Chennai, India; 9 Department of Critical Care, Apollo Speciality Hospitals, Vanagaram, Chennai, India; 10 Institute of Regeneration and Repair, University of Edinburgh, Edinburgh, Scotland, United Kingdom; 11 Critical Care, University College Hospital, London, United Kingdom; 12 Critical Care, Mahidol Oxford Tropical Medicine Research Unit, Bangkok, Thailand; 13 Pandemic Science Hub, University of Edinburgh, Edinburgh, Scotland, United Kingdom

**Keywords:** barriers and facilitators, Consolidated Framework for Implementation Research, critical care, implementation science, low- and middle-income countries, mixed methods, stakeholder engagement

## Abstract

**Introduction:**

Critical care represents one of the highest stakes healthcare environments, which contribute significantly to the global burden of morbidity and mortality. Quality indicators (QIs) are important tools for monitoring and improving the quality of care in intensive care units (ICUs), yet their implementation and evaluation in low- and middle-income countries are insufficiently researched. The aim of this study was to examine the organisational readiness for the implementation of stakeholder-selected ICU QIs and identify the organisational, team, and behavioural factors that influenced the implementation of these QIs in ICUs in India.

**Methods:**

A mixed-methods study was conducted across seven sites (10 ICUs) within the Indian Registry for IntenSive care (IRIS) network between March and October 2023. Organisational readiness was assessed longitudinally using the validated Model for Understanding Success in Quality 2.0 (MUSIQ 2.0) tool at two time points (prior to implementation and 1 year following the first assessment). Barriers and facilitators to implementation were identified through observations of monthly ICU quality improvement meetings, guided by a semi-structured observation framework informed by the Consolidated Framework for Implementation Research (CFIR). A thematic analysis followed Braun and Clarke's six-phase framework, with inductive and deductive coding mapped to CFIR constructs.

**Results:**

Although scores varied across sites, readiness assessments indicated that sites considered themselves ready for quality improvement initiatives. The scores ranged from 118 to 160 in the first assessment and 114–147 during the second assessment, which meant all of the sites had a reasonable chance of success. Successful implementation meant that the sites collected the data for the indicators selected, received feedback of their sites’ performance, and discussed how to use or intend to use the data for service improvement. Four overarching themes shaped implementation success: (1) cognitive bias, (2) leadership and team dynamics, (3) registry usability and implementation support, and (4) organisational resources and staffing. Audit and feedback meetings functioned as the central mechanism through which these factors interacted, enabling collective data interpretation and incremental engagement with quality improvement.

**Conclusions:**

A triad of interdependent conditions, namely, purposeful stakeholder engagement, appropriate resources, and implementation support, determined whether QI data translated into meaningful clinical engagement. These findings have direct implications for the design of registry-based quality improvement initiatives in resource-constrained health systems globally.

## Introduction

Critical care is one of the highest-stakes healthcare environments, where rapid clinical decisions directly impact mortality and morbidity among the most vulnerable patient populations (1, 2). Quality of care (or absence of) is directly linked to patient recovery after critical illness and other metrics such as occupancy, antimicrobial resistance, and cost of care (3, 4). It is therefore essential to systematically monitor the quality of care by using metrics such as quality indicators (QIs), which serve as important measures of structural components, care processes, and patient outcomes in intensive care units (ICUs). The same measures can guide and evaluate service improvement initiatives (5, 6).

QIs widely in use have mostly been validated using higher-income healthcare populations and in relatively homogeneous ICU services such as the UK, Europe, and Canada (7, 8). As a result, these indicators may not be actionable or contextually relevant for implementation in low- and middle-income countries (LMICs) (9). Critical care case mix and antecedent disease burden, together with healthcare infrastructure, workforce capacity, and associated costs of care, although variable both between and within countries, are challenging and more pronounced in LMIC settings, including in India (10). Recognising this gap, expert consensus groups with representation from clinicians, policymakers, and patients are seeking to develop new QIs as well as select appropriate existing QIs that may have priority and validity in the LMIC context (11, 12). In India, the Indian Society of Critical Care Medicine (ISCCM) has framed guidelines for ICU quality indicators, recognising the need for contextualised measurement approaches (13). Despite this progress, there are gaps in understanding how they can be implemented and how well these indicators work in these settings.

Successful implementation of QIs requires understanding the organisational and contextual factors that influence acceptability and utility to inform meaningful care improvements (14, 15). Previous research has identified several barriers to successful implementation, including time constraints, organisational investment, workforce planning, and data knowledge gaps (16). Behavioural factors—such as how individuals and teams engage with data and how they perceive the value of measurement—can further shape whether QIs are used meaningfully or remain as a checkbox measure. Implementation science further reinforces how stakeholder engagement and strategies to increase QI relevance can determine both implementation and utility for quality improvement efforts (17, 18). In LMIC settings where contextual pressures are pronounced and where QI implementation evidence is limited, understanding the factors that enable or hinder their implementation is important to bridge the gap between measurement and meaningful improvement.

The aims of our study were 2-fold: first, to examine the organisational readiness for the implementation of stakeholder-selected ICU QIs in Indian ICUs, and second, to identify the organisational, team, and behavioural factors that influenced the implementation of the QIs in the ICUs. Findings from these lines of enquiry were then integrated to provide a comprehensive account of implementation in this context.

## Materials and methods

### Study design

Our study utilises a mixed-methods design to assess organisational readiness and to elicit clinical teams' perceptions of the factors influencing implementation, and to a lesser extent, how these perceptions may influence the intention to use data for quality improvement. Organisational readiness was assessed at two time points, prior to and 1 year following implementation, using the Model for Understanding Success in Quality 2.0 (MUSIQ 2.0) tool. MUSIQ 2.0 is a validated instrument designed to assess organisational readiness for quality improvement across many domains, including team dynamics, organisational culture, leadership, and external factors specific to the team, ICU, and hospital levels. These domains are relevant in this implementation process, given their established role in both QI measurement and use for clinical improvement (19).

ICU teams' perceptions of the barriers and facilitators to implementation were elicited through observations and field notes that were recorded during ICU multidisciplinary team meetings. A semi-structured observation guide informed by the Consolidated Framework for Implementation Research (CFIR) was used to guide data collection and analysis (14). Refer to Supplementary File 1 for the observation guide used to collect the data.

Findings from this study intend to inform recommendations for future strategies to support site preparation and the implementation of critical care quality indicators in other settings in India.

### Quality indicator selection and implementation in the Indian Registry for IntenSive care network

Eligible quality indicators were selected through a formal stakeholder consensus exercise that has been published previously (11). The selected QIs were then implemented as an optional module available to ICUs that are part of the IRIS. Established in January 2019, IRIS is a cloud-based registry designed to continuously collect standardised clinical data for service evaluation, quality improvement, and clinical research. The IRIS data collection platform was used to collect both core and quality indicator datasets. Details about the registry, the datasets, and data quality assessments have been published elsewhere (20, 21). In brief, the IRIS mandatory minimum core dataset comprises 33 variables collected in the first 24 h of admission and five variables collected on the day of discharge. This core dataset provides information to enable evaluation of ICU case mix, acuity, organ support, and clinical outcomes (21). The QI dataset variables are collected daily after the first 24 h of ICU admission and the process continues through to the day of ICU discharge. Raw and analysed data from both the core and QI datasets are available to each site every month to be downloaded in the form of a report. The report provides information to evaluate temporal and site comparison trends in case mix, outcomes, and quality of care, thus providing a mechanism for audit and feedback to support quality improvement. Published definitions and derivations of the indicators, together with the information to support interpretation of the QIs, are available in Supplementary File 2. An example of the report is provided in Supplementary File 3.

### Site selection

Sites were eligible to participate in the implementation of the selected quality indicators based on three criteria: that they had established mechanisms for daily data collection, there was a dedicated data collector, and core dataset variables captured in the first 24 h of ICU admission had completeness exceeding 95% for at least 1 month prior to implementation. As of 2023–2024 at the time of data collection, the registry included 23 sites, including 55 ICUs with dedicated data collectors. Other desirable criteria were an expressed interest in quality indicator measurement, identification of an ICU champion (a junior doctor or an allied health professional) to liaise with the ICU, and an agreement to participate in monthly meetings to discuss the indicators and the registry report with the implementation team. Each site chose a subset of QIs based on their interest, relevance, and ICU priorities for this mixed-methods evaluation from the wider quality indicator dataset identified through the Delphi consensus exercise (11).

An email was sent to the IRIS registry ICU site leads who were implementing the QIs, inviting them to participate in this mixed-methods evaluation. A participant information sheet informing them about the evaluation and an informed consent form were shared. Any questions and concerns raised were answered by the first author (VP), and an opportunity to speak with registry leads was available.

### Implementation process

Implementation of the quality indicators was led by the IRIS implementation team that comprised of two clinical research associates (CRAs) with expertise in service improvement and the IRIS registry. The CRAs worked together with participating site leads to select their indicators from the stakeholder-prioritised Delphi exercise (11). CRAs conducted training sessions with the data collectors. Training included selection and understanding of the relevant clinical and operational variables to be collected, familiarising data collectors with how to locate and identify source data variables within ICU clinical documentation and troubleshooting and navigation of the IRIS platform. The CRAs were available to the site teams throughout the study for daily online support.

Up to two ICU champions (a junior physician and/or an allied health professional) were identified by the site lead to support them and the data collector in the implementation efforts in their respective ICUs. As a result, each participating site had an ICU team (site lead, data collector, and champion) to lead the QI implementation and monthly meetings, supported by the CRAs. There were 20 participants in total across all the sites, with each site having between two and five members in their ICU teams.

These meetings provided a structured forum for discussing the registry report data (Supplementary File 3), addressing the barriers experienced while implementing the QIs, and exploring team members’ perceptions of the data collected, the QIs, their utility to evaluate the quality of care, and inform future service improvement. The ICU teams gathered in person for these meetings and the CRAs and VP joined virtually using video conferencing software. A description of the implementation processes is available in Supplementary File 4, which is the TIDieR (Template for Intervention Description and Replication) checklist.

### Data collection

MUSIQ 2 assessments were conducted at two time points in each participating site. The first assessment was conducted prior to the start of QI implementation. The second was conducted 1 year later. The assessments were conducted with each of the ICU teams via Zoom video conferencing software where the teams gathered and filled in the MUSIQ 2 tool together in a video call with VP, who provided support when needed. After the conference call, completed assessments were sent to VP by email by one of the team members. All the sites used the same format and followed the same definitions of the terms for the MUSIQ 2.0 assessment. Where possible, the same site-level respondents participated in the two assessments.

Monthly meetings were observed by VP. A total of three meetings were observed at each ICU over the course of 1 year. Each meeting lasted approximately 30–60 min. During and immediately following the meetings, observations were recorded into the relevant sections of the semi-structured observation guide. Reflections in the form of field notes from the CRAs were also collated into the relevant sections of the guide.

Data collection for the QIs, together with the implementation approach, was standardised for all participating sites. All meetings were audio-recorded with prior participant consent. These recordings were referred to during analysis and reflections with the wider research team. Recordings, MUSIQ 2 assessments, and observation notes were pseudonymised and study codes applied to map to sites and saved in a secure drive that was accessible only to the research team.

### Data analysis

#### Assessment of readiness

Responses to the MUSIQ 2 tool were measured on a seven-point Likert scale, with 25 questions grouped into six domains. The domains are QI team, Microsystem, QI support, Organisation, Environment, and Other (19). Assessment resulted in a score between 24 and 168, with higher scores indicating greater readiness to implementation. Scores of 120 and greater indicated a reasonable chance of implementation success, 80–119 indicated possible contextual barriers, and a score of 50–79 indicated serious contextual issues that will likely impede implementation success. The domain-level scores and total scores were reported for each participating site for the two time points.

#### Barriers and facilitators to implementation

The monthly meeting observation notes were analysed using a hybrid thematic analysis approach guided by Braun and Clarke's six-phase framework (22).

The observations and field notes were tagged with pseudonymised site and meeting identifiers to help with systematic coding and cross-site comparisons where needed. Quotations and observation excerpts used to illustrate themes were tagged with the site and meeting number to preserve context and support comparisons across the sites.

Inductive and deductive coding was done side by side. As relevant data segments were identified, they were categorised as barriers or facilitators by way of linking them to representative statements that described the barrier or facilitator experienced. This statement was then mapped to the relevant constructs of the CFIR framework. Statements sharing common mechanisms and context were grouped into broader thematic barrier/facilitator statements that aligned with the relevant CFIR constructs. Coding, data reduction, and theme refinement were done by VP, with synthesis and interpretation supported by three senior researchers (AB, JD, and RH), two of them with experience in qualitative research (AB and RH). NVivo (QSR International) was used for all qualitative data, emergent coding and thematic analysis, and data management.

Observation and field notes were taken using a semi-structured guide made using the CFIR framework allowing for the capture of the relevant information consistently with each ICU team. Data collection was undertaken by the same research team over 1 year fostering the observer–team relationship and facilitating the observation of phenomena such as team dynamics and individual and team lived experiences before, during, and after implementation and data collection. Reflections with more experienced researchers, including those with relevant clinical and contextual experience, provided a structure for reflexivity during analysis and interpretation.

### Ethical considerations

The study and associated materials were approved by the Institutional Ethics Committee—Bio Medical Research, Apollo Hospitals (AMH-C-S-002/01-23), Chennai, and the ethics committee of the University of Birmingham (ERN_2023-0879).

## Results

### Participating ICUs and QIs selected and implemented

Seven sites (10 ICUs) within the Indian Registry for IntenSive care (IRIS) network participated in this evaluation (Table 1). Six sites were located in private hospitals and one in a public hospital. All were mixed medical–surgical ICUs, with bed capacity ranging from 18 to 70 beds. Prior to implementation, all sites met the eligibility criterion and all had achieved 100% completeness of the IRIS core dataset. Implementation of the quality indicators commenced between March and October 2023.

**Table 1:** Site characteristics and QI implementation.

Site characteristics	Site 1	Site 2	Site 3	Site 4	Site 5	Site 6	Site 7
Type of ICU	Mixed	Mixed	Mixed	Mixed	Mixed	Mixed	Mixed
Type of hospital	Private	Private	Private	Private	Private	Private	Public
Number of hospital beds	550	118	300	80	300	150	803
Number of ICUs	3	1	1	1	1	1	2
Number of ICU beds	70	18	25	19	19	20	32
Start of implementation	Aug 2023	Oct 2023	Jun 2023	Jun 2023	Jul 2023	Mar 2023	Aug 2023
Indicators implemented (N)	6	9	7	9	7	6	10
ICU champion available	No	No	No	Yes	Yes	Yes	Yes
Average duration of each meeting (minutes)	29	28	31	50	33	46	50

Four ICUs identified an ICU champion to support implementation activities, while three did not formally designate a champion. All sites received the monthly IRIS registry reports and participated in the planned implementation meetings, with three meetings conducted at each ICU, lasting 28–50 min on average.

Sites selected between 6 and 10 indicators each for implementation. Across all seven sites, 18 unique indicators were implemented in total (Supplementary File 5). There was variation in indicator selection between the sites. “ICU length of stay,” “risk-adjusted ICU mortality (SMR),” “duration of mechanical ventilation,” and “readmission within 48 h of ICU discharge” were selected by all sites, as were indicators reporting antimicrobial stewardship care processes such as “duration of antimicrobial use.” Other indicators were selected by a smaller number of sites, reflecting local clinical priorities and interests, and these included spontaneous breathing trials (four sites); daily Richmond Agitation Sedation Scale (RASS) scoring was also implemented at four sites. These are further described in Supplementary File 5.

### Organisational readiness for implementation

Baseline MUSIQ scores ranged from 118 in Site 1 to 160 in Site 6 (mean 134.6). Six sites scored ≥120 (Sites 2–7), indicating a “reasonable likelihood of implementation success,” while Site 1 scored 118; indicative of contextual barriers (Table 2). Six contextual domains out of 24 consistently scored ≥6 across sites: Organisation QI Culture, QI Team Leadership, QI Team Diversity, QI Team Physician Involvement, QI Team Norms, and QI Team Decision-Making Processes. Greater variation was observed in Organisation Senior Leader Sponsor, QI Team QI Skill, QI Team Prior QI Experience, Microsystem QI Capability, External Motivators, Triggering Event, and Data Infrastructure. Baseline MUSIQ scores indicated that most sites had contextual conditions supportive of implementation, particularly organisational culture, team leadership, and multidisciplinary engagement.

**Table 2:** Average readiness assessment scores (MUSIQ 2): baseline and 1-year follow-up assessment.

Domains of the MUSIQ 2	Site 1	Site 2	Site 3	Site 4	Site 5	Site 6	Site 7
Contextual factors	Average team scores across the two assessments						
External Motivators	1	1	1	1	2.5	6	1
External Project Sponsorship	5.5	6.5	6.5	7	6	3	7
Organisational QI Leadership	5	7	3	4	7	7	5.5
Organisation Senior Leader Sponsor	2.5	0.5	5.5	4	7	7	4
Organisation QI Culture	7	7	6.5	7	7	7	6.5
Organisation QI Maturity	2.5	6.5	6	7	7	7	4
QI Workforce Focus	2.4	4.9	4.3	4.3	5	6.3	6.9
Resource Availability	7	7	6	7	6.5	6.5	6
Data Infrastructure	6	6.5	6.5	3	6	5	5
QI Team Leadership	7	7	7	7	7	7	5.5
QI Team Diversity	6	7	6.5	7	6.5	6.5	5.5
QI Team Subject Matter Expert	6.5	7	7	5.5	7	7	7
QI Team Decision-Making Processes	5.8	6.8	6.8	6.5	6.6	6.6	6.8
QI Team Norms	6	6.9	6.3	6.75	6.9	6.9	6.9
QI Team QI Skill	4	5.5	1.5	4	1.5	7	6
QI Team Physician Involvement	7	7	7	7	7	7	6.5
QI Team Prior QI Experience	3.5	5.5	4	2	6.5	7	5.5
QI Team Tenure	6	4.5	4	7	7	6.5	5.5
Microsystem QI Leadership	5	5	7	3.5	7	6	7
Microsystem Motivation	5.5	5.5	5.5	1	7	5.5	6.5
Microsystem QI Capability	2	4	2.5	2.5	2	5.5	6
Microsystem QI Culture	6	6.5	6.5	7	7	6.5	7
Task Strategic Importance to the Organisation	5	6.5	6.5	7	6.5	7	5.5
Triggering Event	1	1	1	4	7	7	4
Average quality Improvement readiness score (MUSIQ 2 tool) of the Baseline and 12-month scores	***115.63***	***132*** ***.*** ***59***	***124*** ***.*** ***25***	***122.5***	***146.34***	***153.71***	***137.08***

Score	Colour legend
0	Don’t know/NA
1	Totally Disagree
**2**	
**3**	
4	Neither agree nor disagree
5	
6	
7	Totally agree
Total score	Legend
168	Highest possible MUSIQ score
***120–168***	Project has a reasonable chance of success
***80–119***	Project could be successful, but with possible contextual barriers
50–79	Project has serious contextual issues and is not set up for success
25–49	Project should not continue as is. Consider deploying resources to other improvement activities
24	Lowest possible MUSIQ Score when all questions are answered
1	Lowest possible MUSIQ Score (Questions recorded as “don't know” or “N/A”)

Bold italic values denote the overall average MUSIQ 2.0 readiness score per site, as distinct from individual domain scores.

Follow-up scores varied across sites but largely remained within the range associated with a reasonable likelihood of implementation success, suggesting that organisational readiness remained broadly stable during the implementation period. At 1 year, scores ranged from 113 (Site 1) to 147 (Site 6), with the mean score being 131.6. In Site 2, the score increased from 121 to 144, and in one site, it remained unchanged (Site 4). Five sites saw a reduction in scores ranging from 7 to 12 points. ICUs that identified an implementation champion (Sites 4–7) had higher baseline scores (122–160) than those without a champion (Sites 1–3; 118–130), although three of the sites with ICU champions (Sites 5–7) saw a drop in scores in the second assessment by 6–12 points. The only site that had a higher score in the follow-up was one without an ICU champion (Site 2), which reported a 22.83-point increase in the second assessment. The scores from both the assessments are provided in Supplementary File 6.

### Barriers and facilitators to implementation

**Figure 1:**
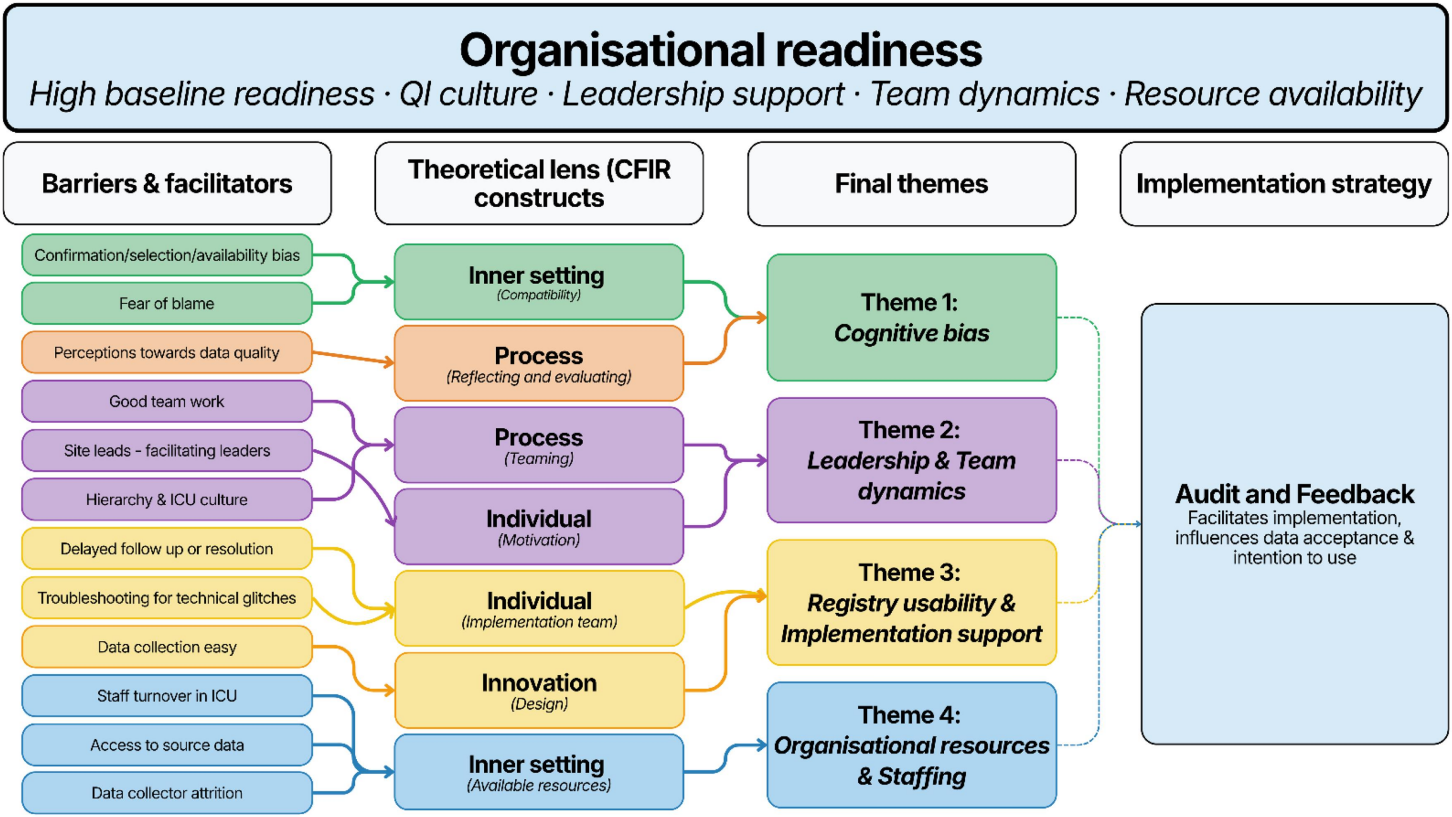
Factors influencing the implementation of quality indicators in ICUs in India.

Twenty-one implementation meetings were conducted across the seven participating sites, generating approximately 13 h of observational data. Four themes were identified from an analysis of observation notes and meeting discussions: (1) cognitive and behavioural biases, (2) team dynamics and leadership, (3) registry usability and implementation support, and (4) organisational resources and staffing (Figure 1).

Informed by constructs from the Consolidated Framework for Implementation Research (CFIR), the emergent themes mapped primarily to CFIR domains of individual characteristics, inner setting, intervention characteristics, and implementation process, reflecting how individual perceptions of the indicators, team dynamics, organisational context, and operational aspects of the registry shaped implementation experiences across sites.

#### Cognitive and behavioural biases

Factors described under this theme relate primarily to the CFIR construct “Inner setting” and “Process,” particularly knowledge and beliefs about the QI indicators within their ICU context and individual responses to the interpretation of data.

During discussions across multiple meetings, clinicians frequently interpreted quality indicator data in relation to their prior beliefs of current ICU care. When reported data appeared consistent with these prior beliefs, it was generally perceived as accurate and credible. In contrast, when indicators (and associated data) appeared inconsistent with clinicians' priors, the clinical team expressed concerns regarding the accuracy of the data or the way the indicators were defined and derived. In three sites (Sites 1, 2, and 7), clinicians also questioned the definitions of certain indicators when these operational definitions did not appear to align with local clinical practices [e.g., culture obtained within 24 h of antibiotic commencement, standardised mortality ratio (SMR), and reintubation within 24 h of extubation]. These patterns suggest confirmation bias and terminology bias influencing the interpretation of the QIs reported and the underpinning data (23, 24).

In several discussions, the ICU team attributed lower QI compliance that suggested poorer quality performance to potential gaps in data entry or limitations in registry reporting. Conversely, when indicators showed favourable performance, these results were more readily interpreted as reflecting actual clinical practice within the ICU. Positive performance was also associated with discussions about expanding monitoring to additional indicators within the registry. Such asymmetries in interpretation are further consistent with confirmation bias in data interpretation (23, 25).

Interpretations of indicator performance were also influenced by recent clinical events discussed during the meetings. In some cases, teams focused on recent cases when interpreting indicator results, even when these events represented a small proportion or “exception” of overall admissions. This tendency to emphasise recent experiences when interpreting performance trends reflected heuristic biases in data interpretation.

Concerns about how indicator performance might be interpreted by hospital management were raised during several meetings, particularly when indicators related to mortality or other outcomes perceived as sensitive. For example, during discussions at Site 2, clinicians reviewing the standardised mortality ratio expressed concern that patient case mix might influence how the ICU's performance would be interpreted:

“…they are very conscious of the mortality and that was not a result of the ICU care and how that it will be reflected as poor performance…”

These discussions indicated that perceptions of organisational accountability and reputation concerns - i.e., fear of blame - influenced the ICU teams' engagement and their intention to use the QI data. This finding maps to the Inner Setting domain of the CFIR, Specifically the Compatibility construct.

Across meetings, the data-driven audit and feedback process, in part enabled by the monthly registry reports, appeared to support continued engagement with the QI data over time. Although some teams maintained a cautious or sceptical stance towards certain indicators (culture obtained within 24 h of antibiotic commencement and SMR that measure avoidable harms or poor care), repeated discussions during meetings were associated with gradual acceptance of the registry data as a source of information for monitoring ICU performance. For example, in the third meeting, the clinician from Site 4, although still unsure of the compatibility of the definition used in the registry, wished to continue monitoring with the acknowledgement that there is the following discrepancy.

“…There was also a clarification on why the antibiotic commencement percentage was not good and that was because of the way the question was phrased in the registry. If the data was entered keeping that in mind, feel that the percentages will be better.”

#### Team dynamics and leadership

Across all participating sites, the QI meetings were typically led by site leads (usually senior clinicians), who guided interpretation of the data and facilitated conversations about how the information might be used within the ICU. The site leads also acted as intermediaries between data collectors and clinical staff, helping to resolve questions related to indicator definitions or data entry processes. The monthly meetings in all sites were chaired by the site leads, who structured the review of the registry reports and moderated team discussions.

Although leadership authority was concentrated with the site leads, meetings in all sites included participation from data collectors and ICU champions (who were nurses in three sites, and physician assistant in one site), who contributed contextual information about clinical processes and data collection practices. In several sites, teams were observed reviewing registry reports together prior to the formal QI meetings. Data collectors described regular communication with the site leads when uncertainties arose during data entry, and in some meetings, the site leads acknowledged the contributions of data collectors to maintaining data completeness and accuracy. These interactions suggested collaborative working relationships between the site leads and the operational staff responsible for data collection.

In some ICUs, discussions during audit and feedback sessions led to consideration of changes in clinical practice. For example, in Site 5, the site lead initiated ward-round discussions and educational activities where a low-performing indicator prompted the site lead monitor to review the indicator during their ward rounds and focused on education and improvement efforts to improve the indicator's compliance. These efforts reflected positively in the subsequent month's performance data reviewed in the next meeting.

These observations suggested that leadership engagement and internal communication structures directly influenced how indicator data were interpreted and teams' intention to use the data for effecting changes within the participating ICUs. Although participation in meetings in all sites included multiple professional roles, decision-making authority regarding how the data would be interpreted and whether changes to clinical processes would be pursued largely remained with the site leads. This pattern reflects the influence of hierarchical clinical structures that exist within ICU teams internationally but perhaps even more so in India, where senior clinicians shape the direction of discussions and subsequent actions. These dynamics correspond to CFIR constructs related to leadership engagement, networks and communication, and the role of key individuals in implementation success.

#### Registry usability and implementation support

Data collectors across sites described the IRIS registry platform as generally straightforward to use, and most reported that data entry could be incorporated into routine workflows. Some technical issues were noted, including delays in patient records appearing on the system or synchronisation between the mobile app and the cloud-based platform. These were typically addressed through communication with the registry team via a dedicated messaging group and were not perceived as major barriers to data collection or QI interpretation.

One data collector without a clinical background described experiencing initial difficulty interpreting clinical variables but reported gaining confidence over time with the support of training materials and guidance from the implementation team.

Support from the implementation team, including clinical research associates (CRAs), was used to clarify indicator definitions and address technical issues raised during meetings. In two sites (Sites 1 and 7), delays in resolving platform and reporting issues limited the teams' ability to access and share indicator reports with the wider ICU staff. For instance, in Site 1, these issues were raised early in the implementation stage and persisted for approximately 8 months, after which they were escalated to the registry team. Although a resolution was reported to be underway, the issues remained unresolved within the study period.

As one site lead noted during a meeting at Site 7:

“…once these issues are fixed, it will be a very valuable report that can be shared with the extended ICU team.”

These observations suggested that both registry usability and the timeliness of technical support influenced teams' ability to review and disseminate indicator data. Delays in accessing reports appeared to affect the acceptability of the registry outputs and teams' intention to share and use the data for quality improvement discussions, corresponding to CFIR constructs related to innovation (design) and implementation support and reinforcing the importance of audit and feedback in the acceptance and utility of the QIs.

#### Organisational resources and staffing

Organisational factors such as resource availability, staffing, and continuity of workforce influenced implementation. Changes in personnel, including turnover of data collectors and leadership transitions, directly disrupted implementation. At Site 6, following the departure of a data collector, and leadership changes and staffing constraints in Sites 3 and 6, implementation slowed and as a result hindered engagement with the emergent data. Several sites also reported difficulties retaining their ICU champions because of competing clinical responsibilities or changes in professional roles.

Some sites described challenges completing data entry during public holidays or weekends when data collectors were absent, particularly when retrieving missing information from hospital medical records departments. Other sites reported responsive support from their medical records departments when similar information was required, which, in turn, influenced perceptions of QI utility, acceptance, and intention to use. These observations highlight the influence of CFIR “inner setting” constructs such as organisational support and available resources. Further information on data quality and performance is published in a separate study and is beyond the scope of this analysis.

## Discussion

### Summary of findings

This mixed-methods evaluation examined factors influencing the implementation of stakeholder-selected quality indicators across seven sites, which included 10 ICUs participating in the IRIS registry in India. Organisational readiness assessed using the MUSIQ-2 tool indicated that most sites had contextual conditions supportive of implementation, including established quality improvement cultures, available resources, and engaged clinical teams. However, qualitative findings demonstrated that favourable organisational readiness did not alone translate into implementation success and sustained intention to use the indicators. Instead, implementation and intention to use was shaped by clinicians' preconceptions of and responses to QIs (performance data), the leadership engagement within ICU teams, registry usability and implementation support, and organisational stability. These findings elevate the importance that behavioural and organisational mechanisms can have in influencing data quality improvement strategies, in addition to the already recognised mechanisms of structural and resource readiness (26).

Mean MUSIQ scores decreased modestly between baseline and follow-up, although sites remained within the category indicating a “reasonable likelihood of implementation success.” This shift likely reflects a transition from aspirational readiness assessed prior to implementation to readiness reassessed after practical experience with implementation activities. Several domains consistently scored below the threshold associated with readiness, “QI workforce focus,” “QI team skill,” and “QI microsystem capability,” which corresponded to operational challenges observed during the study, including operational delays affecting reporting and difficulties maintaining data collection during workforce turnover.

### Beyond readiness

For quality indicators to fulfil their intended purpose, implementation must progress beyond data collection and receiving a monthly report towards active use, whereby teams monitor performance, identify gaps in care and plan service improvements, and show intentions to act on those plans. Although implementation describes the structural aspects of data collection and feedback receipt, meaningful engagement and ultimately intention to use the data requires something beyond structural readiness. Although readiness frameworks identify contextual conditions that may support implementation, they do not capture how clinical teams interpret, trust, and act upon performance data. In this study, clinicians’ engagement with indicator results was shaped by their prior beliefs about care quality, the perceived relevance of indicators to local clinical practice, frustrations with the registry itself (platform usability, reporting issues and delays), and their confidence in the credibility of the data and the perceived risks associated with presenting data that may suggest poor care quality (27–29).

Monthly audit and feedback meetings functioned as spaces where clinicians collectively interpreted registry outputs and negotiated their meaning. Rather than directly triggering practice change, feedback appeared to operate as a sense-making process through which teams evaluated the credibility and relevance of the data before considering action. Similar mechanisms have been described in studies of audit and feedback interventions (23).

These findings are consistent with Clinical Performance Feedback Intervention Theory (CP-FIT), which proposes that feedback effectiveness depends on sequential processes such as perception, acceptance, intention, and behaviour change. Feedback may fail to produce action if any stage of this process stalls. CP-FIT also emphasises that clinicians' existing beliefs about care quality influence how feedback is interpreted (23). Across sites in this study, teams demonstrated varying trajectories, from initial scepticism to sustained engagement with the data, and in some cases, consideration of changes to clinical practice. This suggests that registry-based feedback systems require not only technical infrastructure but also facilitated spaces for collective interpretation and trust building (30, 31).

### Leadership as intermediaries between data and action

Site leads (senior clinicians) played a central role in translating registry outputs into clinically meaningful insights and shaping how the data were used within ICUs. They acted as intermediaries between registry feedback and clinical practice by interpreting results, guiding discussions, and determining when and how information was shared with the wider ICU team. Leadership engagement has previously been identified as a critical determinant of implementation success in healthcare settings (32). In this study, leadership influence also reflected hierarchical clinical structures in which senior clinicians shaped the interpretation of performance data and decisions about practice change. Although this facilitated coordination of implementation activities, it suggests that sustained use of indicator data may depend on broader engagement beyond individual leaders (33).

### Technical infrastructure, implementation support, and workforce stability

Implementation was also influenced by registry usability, CRA support, and workforce stability. Registry platforms that integrate into clinical workflows are more likely to be adopted as quality improvement tools rather than perceived as administrative burdens (21, 34). In this study, delays in resolving reporting issues in two sites limited teams' ability to share indicator outputs with the wider ICU staff, affecting the perceived usefulness and acceptability of the data.

Stable personnel responsible for data collection were also important for maintaining data completeness and continuity of implementation activities. Data collectors functioned not only as technical operators but also as members of the quality improvement team responsible for sustaining the flow of reliable data (35). In settings where staffing turnover is common, workforce instability may therefore directly influence the sustainability of registry-based quality improvement initiatives (36), as such funders may need to consider how to support ongoing sustainability while health systems ingress QI systems and clinical use into their business models.

### Implications for registry-based quality improvement

Together, these findings suggest that successful registry-enabled quality improvement requires alignment between organisational readiness, behavioural engagement with feedback, and operational infrastructure. In this study, audit and feedback meetings acted as the mechanism through which these elements interacted, enabling clinicians to collectively interpret data, address technical barriers, and consider responses to indicator results. Evidence from audit and feedback research indicates that such interventions are most effective when embedded within collaborative learning processes rather than delivered solely through performance dashboards (23, 37). Designing registry-based quality improvement initiatives as facilitated spaces for shared interpretation and learning, perhaps as part of the broader research ecosystem, may therefore strengthen the effectiveness of evidence to practice change in resource-constrained health systems.

### Strengths and limitations

This study has several strengths. It combined longitudinal organisational readiness assessments with inductive and framework analysis of observations of implementation processes, enabling an examination of both contextual readiness and the mechanisms through which implementation unfolded in practice. In addition, the study was conducted across multiple ICUs within a national registry network, allowing a comparison of implementation experiences across sites with differing organisational contexts.

Several limitations should also be considered. Observations were limited to scheduled implementation meetings and may not have captured informal discussions or decision-making processes related to indicator use within the ICUs. Participation was also limited to sites already engaged with the IRIS registry and willing to implement the quality indicators, which may reflect ICUs with greater baseline interest in quality improvement. Finally, the relatively small number of sites within a single national context may limit transferability of the findings to India more widely or to other LMICs. Furthermore, six of the seven participating sites were ICUs situated in multispecialty private hospitals. Although one public hospital was included, the predominantly private sector sample may not represent the resource constraints, workforce patterns, and organisational culture typically found in public sector ICUs in India or other LMIC settings more broadly.

## Conclusion

This study demonstrates that successful implementation of quality indicators in the ICU extends well beyond the provision of data. The conditions necessary for implementation to progress towards quality improvement were shaped by a triad of interdependent conditions: leadership behaviours and purposeful stakeholder engagement, appropriate infrastructure and resources, and sustained implementation support. Critically, clinicians' engagement with the quality indicator data was not simply a function of data availability but was mediated by prior beliefs about care quality, perceived relevance to local practice, and confidence in the plausibility of the data. These findings underscore that quality improvement interventions cannot simply be distributed; rather they must be built upon foundations that are deliberately designed, adequately resourced, and locally adapted to the contexts into which they are introduced.

## Data Availability

The datasets presented in this article are not readily available because the quantitative organisational readiness data supporting the findings of this study are available from the corresponding author upon reasonable request. The qualitative observation data are not publicly available because of confidentiality restrictions and the risk of participant identification given the small number of sites. Requests to access the datasets should be directed to Vrindha Pari, vxp275@student.bham.ac.uk.

## Indian Registry of IntenSive care collaborators

Arunima Dash, All India Institute of Medical Sciences (AIIMS), Bhubaneswar, India, Augustian James, Apollo Hospitals, Chennai, India, Bency John, Apollo Proton Cancer Centre, Chennai, India, Kasturi Sanyal, All India Institute of Medical Sciences (AIIMS), Bhubaneswar, India, Mithilesh Devaraj, Apollo Hospitals, Chennai, India, Mohana Priya Sachidhanandam, Apollo Speciality Hospital, Vanagaram, Chennai, India, Prashant Kumar Pradhan, All India Institute of Medical Sciences (AIIMS), Bhubaneswar, India, Preethika Ravichandran, Apollo First Med, Chennai, India, Rajesh Raghavan, All India Institute of Medical Sciences (AIIMS), Bhubaneswar, India, Revathi Kandeepan, Apollo First Med, Chennai, India, Rohit Aravindakshan Kooloth, Apollo Cancer Speciality Hospital, Chennai, India, Shailaja Kumar, Apollo Speciality Hospital, Vanagaram, Chennai, India, Sowmya Panneer Selvam, Apollo Cancer Speciality Hospital, Chennai, India, Sruthi Mano, Apollo Hospitals, Chennai, India, Swedha Vasudevan, Apollo Speciality Hospital, OMR, Chennai, India, Vijay Vasu, Apollo Cancer Speciality Hospital, Chennai, India, Vinisha Varadhan, Apollo Proton Cancer Centre, Chennai, India.
