# Supplementary material for: Contextual determinants of implementation of stakeholder-selected quality indicators in Indian intensive care units: a mixed-methods evaluation within a national registry network
Source: Front Health Serv. 2026 Jul 27;6:1872641. doi: 10.3389/frhs.2026.1872641 (PMC13454447; doi:10.3389/frhs.2026.1872641)
Supplement: Supplementary file 1 [file Datasheet1.pdf]

Critical Care Asia Africa
CORE Data Completion Guide

Version 1.3 March 2025

Aligned to CCAA data dictionary version 8

Core variables

These variables provide essential information to enable reporting of case mix, acuity, and risk adjusted outcome. The patient identifiers are used to enable individual ICU stakeholders to track patients during the encounter. These are removed during data curation and an encounter ID is generated. All core variables are mandatory by default. Please speak to your national coordinator if data for a variable is not available at your ICU.

On admission

Completed as close to admission of the patient to ICU. Please use information from the time of admission.

Variable name	Data entry format	Dependency	Guidance for collection of variables
**Section: Patient information**			
Patient's name*	Text		The patient’s surname and their given name.
Medical record number*	Text		A number given to a patient for a specific hospital encounter or repeated encounters.
Age*	Number		Age of the patient on the day of admission to the ICU.
Sex*	Dropdown ['Male', 'Female', 'Intersex']		The patient’s sex at birth.
National identity number	Text		A unique identifier for a patient that is given by the government.
Contact number	Number		The phone number of the patient that can be used for contact.
Fee paying	Yes/No		Indicate whether the patient is paying fees.
Fee paying method	Dropdown ['Private Insurance', 'Self/out of pocket', 'Government funded scheme', 'Employer funded/insurance', 'Other']	Fee paying = ‘Yes’	Type of fee paying method.
Other fee paying method	String	Fee paying method = ‘Other’	Specify the other type of fee paying method.
Comorbidities*	Searchable dropdown [see appendix for list of options]		Select conditions currently present and diagnosed prior to this hospital admission. The list of options is mapped to the Charlson Comorbidity Index. Other comorbidities may be important to the clinical team and can be input via the field ‘Comorbidities (Other)’.
Other comorbidity*	Searchable dropdown [Snomed list]	Comorbidities = ‘Other’	Search the SNOMED CT1 list and select any other conditions that are not available in the comorbidities dropdown list.

**Section: Admission details**			
Date of hospital admission*	Date [dd/mm/yyyy]		The date of arrival of the patient in the hospital.
Time of hospital admission*	Time [hh:mm]		The time of arrival of the patient in the hospital.
Date of ICU admission*	Date [dd/mm/yyyy]		The date of arrival of the patient in the ICU.
Time of ICU admission*	Time [hh:mm]		The time of arrival of the patient in the ICU.
ICU admission source*	Dropdown ['Operating theatre - same hospital', 'Operating theatre - other hospital', 'Ward - same hospital', 'Ward - other hospital', 'ED - same hospital', 'ED - other hospital', 'ICU/ HDU - same hospital', 'ICU/ HDU - other hospital']		The patient's clinical location immediately prior to this ICU admission.
Readmission*	Yes/No		This ICU admission encounter is a readmission: defined as; patient was discharged and readmitted to the ICU within the same hospital care episode.
Date of previous discharge	Date [dd/mm/yyyy]	Readmission = 'Yes'	Previous ICU discharge date during the patient's current hospital admission.
Type of admission*	Dropdown ['Non operative', 'Post operative']		Non operative: Patients not arriving directly from the operating theatre or recovery. This includes patients who may have undergone an operative procedure earlier in their hospital encounter.<br><br>Post operative: Patients who have been admitted to the ICU directly from the operation theatre or recovery.
Emergency surgery*	Yes/No	Type of admission = 'Post operative'	Emergency surgery is defined as immediate surgery, where resuscitation (stabilisation and physiological optimisation) is simultaneous with surgical treatment and where surgery normally takes place within minutes of decision to operate.
Reason for admission (Operation)*	Searchable dropdown [Snomed list]	Type of admission = 'Post operative'	Search for the operative procedure from the SNOMED CT1 list which most accurately describes what procedure was undertaken that prompted the ICU admission. If there have been multiple operative procedures, use the additional fields to add additional procedures.

Reason for admission (Disorder)*	Searchable dropdown [Snomed list]	Type of admission = 'Post operative'	In addition to the operative procedure, search and select any disorders from the SNOMED CT list which necessitated admission to the ICU alongside the operation.
Reason for admission (Disorder)	Searchable dropdown [Snomed list]	Type of admission = 'Non operative'	The primary disorder (disease, condition, injury) which has necessitated admission to the ICU. This should be based only on what is known or suspected to be the primary reason for admission. Any additional disorders (disease, conditions or injuries) deemed to be significant to the patient's admission to ICU, identified or presenting following the ICU admission, should be added using the additional reason for admission fields, at any time during the ICU encounter. The chronology of the disorders entered is retained in the data export. The primary disorder known at admission is used to determine severity of illness and prediction of mortality.
Severe acute respiratory infection*	Yes/No		Does the patient have community acquired pneumonia? This variable is used to link with public health surveillance, and disease specific studies.

Assessment (24 hr post admission)

Completed 24 hours after ICU admission. This form reflects events, treatments and assessments occuring in the 24 hour period following ICU admission.

Variable name	Data entry format	Dependency	Guidance for collection of variables
**Section: Respiratory**			
Ventilation*	Dropdown ['Mechanical vent', 'Self vent']		Self vent: No breaths are delivered by a mechanical device during the first 24 hours after ICU admission (this includes breathing by own airway [+/- supplemental oxygen], breathing independently via tracheostomy, and CPAP, HFNC).<br><br>Mechanical vent: All or some of the breaths or a portion of the breaths are delivered by a mechanical device during the first 24 hours after ICU admission.
Route of ventilation*	Dropdown ['Tracheostomy', 'ETT', 'NIV Mask']	Ventilation = 'Mechanical vent'	The type of mechanically assisted breathing during the first 24 hours after ICU admission. If multiple modes are used, please report the

			most invasive (e.g, NIV being the least invasive, and tracheostomy being the most invasive).
Route of ventilation*	Dropdown ['Tracheostomy', 'Own airway']	Ventilation = 'Self vent'	The primary route used by the patient for self-initiated breathing during the first 24 hours after ICU admission. If multiple modes are used, please report the highest support.
Oxygen delivery device*	Dropdown ['High flow oxygen (min flow 30L/min)', 'CPAP', 'Simple face mask', 'Nasal cannula', 'No supplemental oxygen']	Ventilation = 'Self vent'	The oxygen delivery device used by the patient during the first 24 hours after ICU admission. If multiple modes are used, please report the highest support.
PaO_2	Number		The partial pressure of oxygen measured in the arterial blood. The worst recorded value during the first 24 hours after ICU admission.
SpO_2	Number		Percentage of oxygen-saturated haemoglobin corresponding to the worst PaO_2 recorded. If PaO_2 is not recorded, then the worst recorded value during the first 24 hours after ICU admission.
FiO_2	Number		The inspired oxygen concentration delivered at the time of the worst PaO_2 recorded. If PaO_2 is not recorded, then enter the inspired oxygen concentration recorded at the time of the worst SpO_2.
$PaCO_2$	Number		The partial pressure of carbon dioxide measured in the arterial blood. The worst recorded value during the first 24 hours after ICU admission.
Arterial pH	Number		The measurement of the pH of plasma of an arterial blood sample. The most deranged reading recorded during the first 24 hours after ICU admission. This is the reading furthest from the normal range of pH 7.35 to 7.45.
Respiratory rate*	Number		Worst recorded respiratory rate during the first 24 hours after ICU admission.
**Section: Cardiovascular System**			
Heart rate*	Number		Worst recorded heart rate during the first 24 hours after ICU admission.
Systolic blood pressure*	Number		Worst recorded systolic blood pressure during the first 24 hours after ICU admission
Diastolic blood pressure*	Number		Worst recorded diastolic blood pressure during the first 24 hours after

			ICU admission
Temperature*	Number		Worst recorded temperature during the first 24 hours after ICU admission.
Cardiovascular support*	Yes/No		The use of continuous intravenous vasoactive medication during the first 24 hours after ICU admission.
Vasoactive therapy*	Dropdown ['Dopamine ≤5 or Dobutamine (any dose)', 'Dopamine >5, Epinephrine ≤0.1, or Norepinephrine ≤0.1', 'Dopamine >15, Epinephrine >0.1, or Norepinephrine >0.1', 'Epinephrine >0.3 or Norepinephrine >0.3']	Cardiovascular support = 'Yes'	The type and dose of vasoactive drug used from the list of options.
**Section: Neurological**			
Sedated*	Yes/No		Use of sedative drugs for a minimum of one hour continuous infusion, or greater than one bolus.
Glasgow coma scale (eye)*	Dropdown ['No eye opening', 'Eye opening in response to pain', 'Eye opening to speech', 'Eye opening spontaneously']		The worst (lowest) recorded GCS during the first 24 hours after ICU admission. If the patient is sedated, and or mechanically ventilated then it is the score immediately prior to sedation/ ventilation.
Glasgow coma scale (verbal)*	Dropdown ['None', 'Incomprehensible sounds', 'Inappropriate words', 'Confused', 'Oriented']		The worst (lowest) recorded GCS during the first 24 hours after ICU admission. If the patient is sedated, and or mechanically ventilated then it is the score immediately prior to sedation/ ventilation.
Glasgow coma scale (motor)*	Dropdown ['No motor response', 'Extension to pain', 'Flexion in response to pain', 'Withdraws from pain', 'Localizes to pain', 'Obeys commands']		The worst (lowest) recorded GCS during the first 24 hours after ICU admission. If the patient is sedated, and or mechanically ventilated then it is the score immediately prior to sedation/ ventilation.
AVPU Scale	Dropdown ['Alert', 'Verbal', 'Pain', 'Unresponsive', 'Sedated']		The worst recorded AVPU during the first 24 hours after ICU admission. If the patient is sedated, and or mechanically ventilated then it is the score immediately prior to sedation/ ventilation. [*We recommend using GCS as the primary variable to measure conscious level as this score enables derivation of severity of illness. If GCS is not measured as standard practice in the ICU, AVPU scale may be used as an alternative. If selected, mandatory status should be adjusted accordingly.*]

			Messages addressed to "meeting group chat" will also appear in the meeting group chat in Team Chat
**Section: Renal / Genitourinary**			
Renal replacement therapy*	Yes/No		Use of renal replacement therapy during the first 24 hours after ICU admission. Any duration of RRT is considered as a ‘Yes’.
**Section: Laboratory Results**			
Haemoglobin	Number		The lowest recorded haemoglobin measured during the first 24 hours after ICU admission. If unavailable, provide the haemoglobin reported prior to admission (max 24 hrs prior).
Platelet count	Number		The lowest recorded platelet count during the first 24 hours after ICU admission. If unavailable, provide the platelet count reported prior to admission (max 24 hrs prior).
Packed cell volume	Number		Also called the haematocrit. This is the volume percentage of red blood cells (RBC) in blood. Please record the lowest value during the first 24 hours after ICU admission. If unavailable, provide the PCV reported prior to admission (max 24 hrs prior).
Serum sodium	Number		The worst blood sodium recorded during the first 24 hours after ICU admission. If unavailable, provide the blood sodium reported prior to admission (max 24 hrs prior).
Serum potassium	Number		The worst blood potassium during the first 24 hours after ICU admission. If unavailable, provide the blood potassium reported prior to admission (max 24 hrs prior).
Serum HCO3	Number		The worst blood bicarbonate recorded during the first 24 hours after ICU admission. If unavailable, provide the blood bicarbonate reported prior to admission (max 24 hrs prior).
Serum creatinine	Number		The worst blood creatinine measured during the first 24 hours after ICU admission. If unavailable, provide the last reported blood creatinine prior to admission (max 24 hrs prior).
Serum bilirubin	Number		The worst blood bilirubin measured during the first 24 hours after ICU admission. If unavailable, provide the last reported blood bilirubin prior to admission (max 24 hrs prior).

Blood urea	Number		The worst (highest) blood urea recorded between ICU admission and midnight. If unavailable, the blood urea reported prior to admission (max 24 hrs prior).
White blood cell count	Number		The worst WBC recorded during the first 24 hours after ICU admission. If unavailable, provide the WBC reported prior to admission (max 24 hrs prior)
**Section: Infection**			
Antimicrobial use*	Yes/No		Use of antimicrobial therapy during the first 24 hours after ICU admission. Even if only one dose has been administered this is considered as a 'Yes'.
Antimicrobial type*	Dropdown ['Acyclovir', 'AKT4 <TB>', 'Amikacin', 'Amoxicillin', 'Amphotericin-B', 'Ampicillin/Sulbactam', 'Ampicillin', 'Anidulafungin', 'Antifungal', 'Artesunate', 'Augmentin', 'Azithromycin', 'Aztreonam', 'Caspofungin', 'Cefazolin', 'Cefepime', 'Cefixime', 'Cefoperazone', 'Cefoperazone/Sulbactum', 'Cefotaxime', 'Ceftazidime', 'Ceftriaxone', 'Cefuroxime', 'Chloramphenicol', 'Ciprofloxacin', 'Clarithromycin', 'Clavulanic acid', 'Clindamycin', 'Cloxacillin', 'Co-amoxiclav', 'Coartem', 'Colistin <polymixin E>', 'Cotrimoxazole', 'Dicloxacillin', 'Doxophylline', 'Doxycycline', 'Ertapenem', 'Erythromicin', 'Ethambutol', 'Favipiravir', 'Flucloxacillin', 'Fluconazole', 'Fosfomycin', 'Gancyclovir', 'Gentamicin', 'Gentamycin', 'Imipenem', 'Imipenem/Cilastatin', 'Isoniazid', 'Ivermectin', 'Levofloxacin', 'Linezolid', 'Meropenem', 'Methicillin', 'Metronidazole <Flagyl> ', 'Micafungin', 'Minocycline', 'Moxifloxacin', 'Nafcillin', 'Nitrofurantoin', 'Norfloxacin', 'Other', 'Penicillin',	Antimicrobial use = 'Yes'	Select the type of antimicrobial from the list provided.

	'Piperacillin/Tazobactam', 'Polymyxin', 'Pyrazinamide', 'Remdesivir', 'Rifampicin', 'Rifaximin', 'Teicoplanin', 'Ticarcillin', 'Ticarcillin clavulanic acid', 'Tigecycline', 'Timentin', 'Tobramycin', 'Trimethoprim/Sulfamethoxazole', 'Vancomycin', 'Voriconazole']		
Other antimicrobial type*	String	Antimicrobial type = 'Other'	Specify the other type of antimicrobial administered.

ICU outcome

Completed following ICU discharge.

Variable name	Data entry format	Dependency	Guidance for collection of variables
Date of ICU discharge*	Date [dd/mm/yyyy]		The date on which the patient is discharged from or dies in the ICU.
Time of ICU discharge*	Time [hh:mm]		The time at which the patient is discharged from or dies in the ICU.
**Section: Clinical outcomes**			
ICU discharge status*	Dropdown ['Alive', 'Dead']		Whether the patient is alive at the point of ICU discharge.
ICU discharge destination*	Dropdown ['Home', 'Other ICU', 'HDU', 'Ward', 'Transfer for specialist care', 'Discharge home for end of life care', 'Other hospital', 'Other', 'Nursing home or long-term care facility', 'Rehabilitation hospital']	ICU discharge status = 'Alive'	Indication of the location the patient is destined for immediately after ICU discharge.
Decision to discharge*	Dropdown ['Left against medical advice', 'Patient request', 'Clinician decision']	ICU discharge status = 'Alive'	Specify who made the decision to discharge the patient.
Treatment limitations during ICU stay*	Dropdown ['Withdrawal of treatment', 'Limitation of treatment', 'No limitation']	ICU discharge status = 'Alive'	Indicate whether there was withdrawal of or limitations on treatment.
Type of treatment limitation	Dropdown ['Not for intubation', 'Not for	Treatment	Select the type of treatment limitation.

during ICU stay*	renal replacement therapy', 'Not for ICU readmission', 'Not for vasopressors']	limitations = ‘’Limitation of treatment’’	
**Section: Intervention**			
CPR performed *	Yes/No		Score if the patient has had CPR (heart massage) during the ICU episode of care. Cardioversion without heart massage does not apply as CPR.
**Section: Other**			
Discharge note	String		Any notes about the patient at the time of discharge or any post-discharge care instructions.

Hospital outcome

Completed following hospital discharge.

Variable name	Data entry format	Dependency	Guidance for collection of variables
Date of hospital discharge*	Date [dd/mm/yyyy]		The date on which the patient is discharged from-, or dies in the hospital.
Time of hospital discharge*	Time [hh:mm]		The time at which the patient is discharged from-, or dies in the hospital.
**Section: Clinical outcomes**			
Hospital discharge status*	Dropdown ['Alive', 'Dead']		Whether the patient is alive at the point of hospital discharge
Hospital discharge destination*	Dropdown ['Home', 'ICU', 'Transfer for specialist care', 'Discharge home for end of life care', 'Nursing home or long-term care facility', 'Rehabilitation hospital', 'Transfer to another acute hospital', 'Other']	Hospital discharge status = ‘Alive’	Indication of the location the patient is destined for immediately after hospital discharge.

Appendix

Risk predictions in APACHE II are based on:

The APACHE II score – a score from 0 to 71 consisting of weights for age at admission to your unit (0 to 6 points) and severe conditions in the past medical history (0 to 5 points) plus an Acute Physiology Score (0 to 60 points) based on weightings for deviations from normal in the following twelve physiological parameters during the first hour in the unit. In the absence of information available in the first hour, information from the first 24hrs is used.

o temperature
o mean arterial pressure
o heart rate
o respiratory rate
o A-aDO2 (if FiO2 ≥ 0.5) or PaO2 (if FiO2 < 0.5)
o arterial pH (or serum bicarbonate if no arterial blood gas recorded)
o serum sodium
o serum potassium
o serum creatinine (with double weighting for acute renal failure)
o haematocrit (estimated from haemoglobin
o white blood cell count
o Glasgow Coma Score (assumed to be normal for patients sedated or paralysed and sedated for the whole of the first 24 hours in the unit, or for the entire stay if less than 24 hours)
Admission directly from theatre following emergency surgery
Diagnostic category (weightings for 58 non-surgical diagnoses and 50 surgical diagnoses, plus seven body systems, and a weighting for CPR within 24 hours prior to admission that overrides any other diagnostic category)

Exclusions

Admissions are excluded from the calculation of the APACHE II score if:
a. age at admission to your unit is less than 16 years; or
b. length of stay in your unit is less than 8 hours.

Additionally, admissions are excluded from the calculation of an APACHE II risk prediction if:
c. the admission is for primary burns;
d. the admission is following coronary artery bypass graft (CABG) surgery;
e. the admission is transferred in from another ICU; or
f. all twelve physiological variables are missing.
Readmissions of the same patient within the same hospital stay and admissions missing ultimate hospital outcome are excluded from comparisons of observed and expected mortality

Risk predictions in E-TropICS are based on:

The E-TropICS score consists of 5 physiological variables measured on admission to the ICU and 2 laboratory values.

- Temperature
- Heart rate
- GCS
- Systolic Blood pressure
- Mechanically ventilated
- Hemoglobin
- Blood urea

SOFA severity scores are based on:

The SOFA score consists of 6 organ system related variables measured on admission to the ICU.

- PaO_2/FiO_2
- Platelet count
- Serum bilirubin
- Vasoactive therapy
- Systolic blood pressure
- Diastolic blood pressure
- GCS
- Serum creatinine

Variables required for the calculation of quality indicators

1. Early unplanned ICU readmission rate
    - Date of ICU admission
    - Readmission
    - Date of previous discharge
2. Length of ICU stay
    - Date of ICU admission
    - Date of ICU discharge

Definitions for calculation of quality indicators

Indicator	Reference	Definition
Early unplanned ICU readmission rate	doi:10.1097/MLR.0b013e318293c2fa	Percentage of patients with an unplanned readmission to ICU within 48 hours of ICU discharge during the same hospitalization, per 100 patients eligible for readmission at point of discharge alive from ICU. Excludes: patients discharged from ICU for palliation or to die at home, and patients

		transferred to another ICU or another hospital.<br>Numerator: total unplanned ICU readmissions within 48 hours of ICU discharge during the same hospital encounter. Excludes scheduled ICU admissions following elective procedures.<br>Denominator: Total patients eligible for ICU readmission upon discharge alive from ICU.
Length of ICU stay	doi:10.1097/01.CCM.0000240233.01711.D9	Median (IQR) number of days patients spend in the ICU. Measured per episode of ICU care. Calculated using the interval (measured in hours) between the date and time of ICU admission and the date and time of ICU discharge. Rounded to the nearest 1 decimal place.
Occupancy	doi:10.1097/MLR.0b013e318293c2fa	Number of patients in the ICU in a given 24-hour period for at least 2 hours, standardized for each ICU and each year.
ICU turnover	doi:10.1164/rccm.201304-0622OC	Percentage of newly admitted patients to the ICU in the prior 24 hrs. Numerator: all admissions admitted or readmitted to the ICU in the preceding 24 hours. Denominator = total **potential** occupancy (=capacity) total available unoccupied ICU beds
Comorbidities		['AIDS', 'Acute leukemia', 'Angina', 'Aplastic anemia', 'Arrhythmia', 'Asthma', 'Bronchiectasis', 'Cardiovascular diseases', 'Cerebrovascular disease', 'Chronic hematologic disease', 'Chronic lymphocytic leukemia', 'Chronic pulmonary disease', 'Cirrhosis', 'CKD requiring dialysis', 'Congestive heart failure', 'Connective tissue disease', 'Current gestational diabetes', 'Dementia', 'Depression', 'Epilepsy', 'GI bleeding', 'Hemiplegia <paraplegia>', 'Hepatic disease, Mild', 'Hepatic disease, Moderate to severe', 'HIV', 'Hyperlipidemia', 'Hypertension', 'Inflammatory bowel disease', 'Interstitial lung disease', 'Leukemia', 'Lymphoma', 'Malignant neoplasm', 'Malnutrition', 'Metastatic cancer', 'Myelodysplastic syndrome', 'Myeloma', 'Myocardial infarction', 'Non diabetic endocrine', 'Obesity', 'Other immunosuppressive disease', 'Other neurological condition', 'Peptic ulcer', 'Peripheral vascular disease', 'Pharyngeal neuromuscular', 'Primary lung cancer', 'Primary or inherited immune deficiency', 'Renal failure, Mild', 'Renal failure, Moderate to severe', 'Respiratory disease, Mild', 'Respiratory disease, Severe-moderate', 'Rheumatological condition', 'Secondary immunodeficiency syndromes', 'Severe chronic neutropenia', 'Sickle cell', 'Transient ischemic attack', 'Tuberculosis', 'Tumor', 'Type 1 Diabetes', 'Type 1 diabetes with complications', 'Type 2 Diabetes', 'Type 2 diabetes with complications', 'Valve disease', 'Other', 'None']

Critical Care Asia Africa
QI Data Completion Guide

Version 1.2 14th February 2025
Aligned to CCAA data dictionary version 7

QI variables

Daily form

Completed daily from the day after ICU admission up to, and including the day of ICU discharge. Each daily form reflects events on that date from 00:00 to 24:00.

Variable name	Data entry format	Dependency	Guidance for collection of variables
Date*	Date [dd/mm/yyyy]		Record the date for which the form has been filled. Eg. if the form is completed on the 02/01/2024 for events taking place between 00:00 to 24:00 on the 01/01/2024, the date 01/01/2024 should be recorded.
**Section: Respiratory**			
Ventilation*	Dropdown ['Mechanical vent', 'Self vent']		Self vent: No breaths are delivered by a mechanical device for a period of one hour during the day (this includes breathing by own airway [+/- supplemental oxygen], breathing independently via tracheostomy, and CPAP, HFNC).<br>Mechanical vent: All or some of the breaths or a portion of the breaths are delivered by a mechanical device for one hour during the day.
Route of ventilation*	Dropdown ['Tracheostomy', 'ETT', 'NIV Mask']	Ventilation = 'Mechanical vent'	The type of mechanically assisted breathing for at least a period of one hour during the day. If multiple modes are used, please report the most invasive (e.g, NIV being the least invasive, and tracheostomy being the most invasive).
Route of ventilation*	Dropdown ['Tracheostomy', 'Own airway']	Ventilation = 'Self vent'	The primary route used by the patient for self-initiated breathing during the day. If multiple modes are used, please report the highest support.
Oxygen delivery device*	Dropdown ['High flow oxygen (min flow 30L/min)','CPAP', 'Simple face mask', 'Nasal cannula', 'No supplemental oxygen']		The oxygen delivery device used by the patient. If multiple modes are used, please report the highest support.
Lowest FiO_2*	Number	Ventilation = 'Mechanical vent'	Enter the lowest FiO2 recorded on the ICU flowchart on this calendar day, while the patient is invasively mechanically ventilated. Do not include FiO2 data from periods when the patient was not invasively ventilated.

Lowest SpO_2*	Number		The lowest arterial oxygen saturation measured by pulse oximetry.
Lowest PEEP*	Number	Ventilation = ‘Mechanical vent’	Lowest positive end expiratory pressure for a minimum of one hour.
Highest respiratory rate*	Number		The highest respiratory rate recorded.
Type of lung radiology investigation*	Dropdown ['CXR', 'CT', 'US', 'Other', 'None']		Please record the type of lung radiology investigation performed today. Select ‘None’ if no investigation was performed.
Findings of infiltrates on CXR*	Yes/No	Type of lung radiology investigation = ‘CXR’	Record if infiltrates were found on CXR.
Findings of infiltrates on CT*	Yes/No	Type of lung radiology investigation = ‘CT’	Record if infiltrates were found on CT.
**Section: Cardiovascular System**			
Highest temperature*	Number		Highest recorded temperature.
Cardiovascular support*	Yes/No		Use of continuous intravenous vasoactive medication for a minimum period of one hour.
Vasoactive therapy*	Dropdown ['Dopamine ≤5 or Dobutamine (any dose)', 'Dopamine >5, Epinephrine ≤0.1, or Norepinephrine ≤0.1', 'Dopamine >15, Epinephrine >0.1, or Norepinephrine >0.1', 'Epinephrine >0.3 or Norepinephrine >0.3']	Cardiovascular support = ‘Yes’	The type and dose of vasoactive drug used from the list of options. The patient must have received that level of support for at least one hour.
Central venous catheter*	Dropdown ['New', 'Insitu', 'None']		The presence of a central venous catheter.
Central venous catheter site*	Dropdown	Central venous catheter = ‘New’ or ‘Insitu’	Select the location of the new or existing central venous catheter.
Arterial catheter*	Dropdown ['New', 'Insitu', 'None']		The presence of an arterial catheter
**Section: Neurological**			
Sedated*	Yes/No		Use of sedative drugs for a minimum of one hour. If bolus sedation,

			then 2 or more bolus.
Spontaneous awakening trial (SAT)*	Yes/No	Sedated = ‘Yes’	Spontaneous awakening trial (SAT or “sedation hold”) a period during which sedating medications that are being used to treat the patient were held in order to determine whether the patient requires ongoing sedation or can be managed without sedatives.
Target RASS score*	Dropdown ['-5', '-4', '-3', '-2', '-1', '0', '1', '2', '3', '4', 'No target set']	Ventilation = ‘Mechanical vent’ and Sedated = ‘Yes’	The target RASS is a measure of sedation, awakeness and agitation. The target RASS is the target set each day on the ward round.
Actual RASS score*	Dropdown ['-5', '-4', '-3', '-2', '-1', '0', '1', '2', '3', '4', 'No target set']	Ventilation = ‘Mechanical vent’ and Sedated = ‘Yes’	The RASS is a measure of sedation, awakeness and agitation. The actual RASS is the measured (observed) `RASS either immediately prior to a SAT or at 0800.
**Section: Renal / Genitourinary**			
Urinary catheter*	Dropdown ['New', 'Insitu', 'None']		The presence of a urinary catheter.
Haemodialysis catheter*	Dropdown ['New', 'Insitu', 'None']		The presence of a haemodialysis catheter.
Haemodialysis catheter site*	Dropdown ['IJV', 'Sub clavian', 'Femoral']	Haemodialysis catheter = ‘New’ or ‘Insitu’	Select the location of the new or existing haemodialysis catheter.
Renal replacement therapy*	Yes/No		Use of renal replacement therapy. Any duration of renal replacement therapy is considered as ‘Yes’. Includes RRT administered outside the ICU while they were an ICU patient (e.g. RRT given in a dialysis unit).
Renal replacement therapy type*	Dropdown ['Continuous', 'Intermittent']	Renal replacement therapy = ‘Yes’	The type of renal replacement therapy used.
**Section: Events**			
Inadvertent/ accidental extubation*	Yes/No		The unexpected or unplanned extubation of a patient.
Spontaneous breathing trial (SBT)*	Yes/No	Route of ventilation (mechanical) = ‘Tracheostomy’ or ‘ETT’	A trial of spontaneous breathing, defined as one of the following four;<br>- Trial of ‘T’ piece<br>- Pressure support with low support (PS of 5-8)<br>- CPAP (i.e. only PEEP between 0-5)<br>- Automatic tube compensation with CPAP

			Select 'No' if not eligible for SBT.
Head of Bed >30° or patient sat up*	Yes/No	Ventilation = 'Mechanical vent'	Please record HoB position daily based on observed elevation at time of review.<br>If no degree measure patients have hip flexion with chest and head elevated using pillows or have total bed tilt (Trendelenburg) .
Pressure sore*	Yes/No		New pressure/ compression related injury to skin. New is defined as occurring within the ICU.
Site of pressure sore*	Dropdown ['Foot', 'Ankle', 'Lower leg-front', 'Lower leg-back', 'Buttocks', 'Sacrum', 'Lower back', 'Upper back', 'Shoulder', 'Upper arm', 'Forearm', 'Hand', 'Head/Scalp']	Pressure sore = 'Yes'	Select site of pressure sore from the dropdown list.
Grade of pressure sore*	Dropdown ['Grade 1: Non-blanchable erythema of intact skin', 'Grade 2: Partial thickness skin loss involving epidermis, dermis, or both', 'Grade 3: Full thickness skin loss involving damage to or necrosis of subcutaneous tissue', 'Grade 4: Extensive destruction, tissue necrosis or damage to supporting structures']	Pressure sore = 'Yes'	Select the grade of pressure sore from the dropdown list.
**Section: Infection**			
Highest white blood cell count	Number		The highest recorded serum white blood cell count.
Lowest white blood cell count	Number		The lowest recorded serum white blood cell count.
Antimicrobial use*	Yes/No		Use of antimicrobial therapy. Even if only one dose has been administered, this is 'Yes'
Antimicrobial type*	Dropdown ['Acyclovir', 'AKT4 <TB>', 'Amikacin', 'Amoxicillin', 'Amphotericin-B', 'Ampicillin/Sulbactam', 'Ampicillin', 'Anidulafungin', 'Antifungal', 'Artesunate', 'Augmentin', 'Azithromycin', 'Aztreonam', 'Caspofungin', 'Cefazolin', 'Cefepime', 'Cefixime', 'Cefoperazone',	Antimicrobial use = 'Yes'	Select the type of antimicrobial from the dropdown list.

	'Cefoperazone/Sulbactum', 'Cefotaxime', 'Ceftazidime', 'Ceftriaxone', 'Cefuroxime', 'Chloramphenicol', 'Ciprofloxacin', 'Clarithromycin', 'Clavulanic acid', 'Clindamycin', 'Cloxacillin', 'Co-amoxiclav', 'Coartem', 'Colistin <polymixin E>', 'Cotrimoxazole', 'Dicloxacillin', 'Doxophylline', 'Doxycycline', 'Ertapenem', 'Erythromicin', 'Ethambutol', 'Favipiravir', 'Flucloxacillin', 'Fluconazole', 'Fosfomycin', 'Gancyclovir', 'Gentamicin', 'Gentamycin', 'Imipenem', 'Imipenem/Cilastatin', 'Isoniazid', 'Ivermectin', 'Levofloxacin', 'Linezolid', 'Meropenem', 'Methicillin', 'Metronidazole <Flagyl> ', 'Micafungin', 'Minocycline', 'Moxifloxacin', 'Nafcillin', 'Nitrofurantoin', 'Norfloxacin', 'Other', 'Penicillin', 'Piperacillin/Tazobactam', 'Polymyxin', 'Pyrazinamide', 'Remdesivir', 'Rifampicin', 'Rifaximin', 'Teicoplanin', 'Ticarcillin', 'Ticarcillin clavulanic acid', 'Tigecycline', 'Timentin', 'Tobramycin', 'Trimethoprim/Sulfamethoxazole', 'Vancomycin', 'Voriconazole']		
Other antimicrobial type*	String	Antimicrobial type = ‘Other’	Specify the other type of antimicrobial.
Antimicrobial indication*	Dropdown ['Medical prophylaxis', 'Surgical prophylaxis', 'Treatment of community infection', 'Treatment of hospital infection', 'Treatment of long term care facility infection', 'Other', 'Unknown']	Antimicrobial use = ‘Yes’	Select the indication for antimicrobial use from the dropdown list.
Other antimicrobial indication*	String	Antimicrobial indication = ‘Other’	Specify the other indication for antimicrobial use.

Suspected infection source*	Dropdown ['Bacteremia', 'Gastrointestinal tract', 'Respiratory tract', 'Skin, soft tissue, bone, joint', 'Systemic', 'Urinary tract', 'Other', 'Unknown']	Antimicrobial indication = 'Treatment of community infection' or 'Treatment of hospital infection' or 'Treatment of long term care facility infection'	Select the suspected source of community/hospital/long term care facility infection from the dropdown list.
Other suspected infection source*	String	Suspected infection source = ‘Other’	Specify the other suspected source of community/hospital/long term care facility infection.
Specimen obtained today*	Yes/No		Was a specimen taken today?
Type of specimen*	Dropdown ['Blood', 'Urine', 'CSF', 'Stool', 'Sputum', 'BAL', 'Wound exudate', 'Pleural fluid', 'Peritoneal fluid', 'Pericardial fluid', 'Tracheal aspirate', 'Fluid from infected collection (Other)', 'Throat/nose swab', 'Tissue', 'Indwelling catheter tip', 'Synovial fluid']	Specimen obtained today = ‘Yes’	Type of specimen taken today.
Specimen report*	Yes/No		Record reports for all specimens returned today
Date specimen taken*	Date [dd/mm/yyyy]	Specimen report = ‘Yes’	Record the date the specimen was taken.
Type of specimen*	Dropdown ['Blood', 'Urine', 'CSF', 'Stool', 'Sputum', 'BAL', 'Wound exudate', 'Pleural fluid', 'Peritoneal fluid', 'Pericardial fluid', 'Tracheal aspirate', 'Fluid from infected collection (Other)', 'Throat/ nose swab', 'Tissue', 'Indwelling catheter tip', 'Synovial fluid']	Specimen report = ‘Yes’	Record the type of specimen taken.
Type of test performed*	Dropdown ['Culture and sensitivity', 'Immunoassay (PCR)', 'Immunoassay (other)', 'Serology (AFB)', 'Serology (IgG and IgM)', 'Serology (antigen)', 'Serology (HIV)', 'Serology (other)']	Specimen report = ‘Yes’	Record the type of test performed from the dropdown options.

Test result*	Dropdown ['Positive / Growth', 'Negative / No growth']	Specimen report = ‘Yes’	Whether the specimen report result was positive or negative.
Organism*	Dropdown [see appendix for the list of options]	Type of test performed = ‘Culture and sensitivity' and Test result = 'Positive / Growth'	Select from the list the organisms identified in the specimen report.
Antimicrobial resistance	Dropdown [see list provided for antimicrobial type]	Organism is recorded	From the dropdown list, record the antimicrobial resistance for the organism.
Antimicrobial sensitivity	Dropdown [see list provided for antimicrobial type]	Organism is recorded	From the dropdown list, record the antimicrobial sensitivity for the organism.
**Section: Intervention**			
Venous thromboembolism (VTE) prophylaxis*	Dropdown ['Mechanical', 'Pharmacological', 'Both', 'None']	Ventilation = ‘Mechanical vent’	Use of mechanical or pharmacological prophylaxis in the prevention of venous thromboembolism.
Venous thromboembolism (VTE) prophylaxis contraindication*	Dropdown ['Active bleeding (eg. gastrointestinal bleed, cerebrovascular accident)', 'Bleeding risk (eg. post surgery, active labour)', 'Patient refusal', 'Already on anticoagulation medication', 'Deranged clotting']	Stress ulcer prophylaxis = ‘None’	A contraindication preventing the use of mechanical and pharmacological prophylaxis.
Stress ulcer prophylaxis*	Yes/No	Ventilation = ‘Mechanical vent’	A drug is prescribed for the purpose of stress ulcer prophylaxis.
Stress ulcer prophylaxis drug*	Dropdown ['Cimetidine', 'Dexlansoprazole', 'Esomeprazole', 'Famotidine', 'Lansoprazole', 'Nizatidine', 'Omeprazole', 'Pantoprazole', 'Rabeprazole', 'Ranitidine', 'Sucralfate']	Stress ulcer prophylaxis = ‘Yes’	The named drug from the provided list used for stress ulcer prophylaxis.
Physiotherapy/ mobilisation*	Dropdown ['On bed PT', 'Out of bed PT', 'No PT delivered', 'Patient not eligible']		Patients ventilated may receive passive or active physiotherapy in the ICU. The type of physiotherapy provided should be recorded daily. The event may occur at any time in the previous 24 hours. Some patients may not be eligible for daily physio (for example due to cardiovascular instability, or because they are at the end of life or because they are receiving a procedure).

Appendix

Organism list:

['A/H3N2', 'Acanthamoeba spp.', 'Achromobacter spp.', 'Acinetobacter baumannii', 'Acinetobacter calcoaceticus', 'Acinetobacter haemolyticus', 'Acinetobacter lwoffii', 'Acinetobacter spp., other', 'Actinomyces spp.', 'Adenovirus', 'Aeromonas spp.', 'Agrobacterium spp.', 'Alcaligenes spp.', 'Alpha Coronavirus', 'Ancylostoma brazilense', 'Ancylostoma caninum', 'Ancylostoma ceylanicum', 'Ancylostoma duodenale', 'Angiostrongylus cantonensis', 'Angiostrongylus costaricensis', 'Anncaliia spp.', 'Ascaris lumbricoides', 'Ascaris suum', 'Aspergillus fumigatus', 'Aspergillus niger', 'Aspergillus spp., other', 'Babesia spp.', 'Bacillus spp.', 'Bacteroides fragilis', 'Bacteroides other', 'Balamuthia mandrillaris', 'Balantidium coli', 'Baylisascaris procyonis', 'Beta Coronavirus', 'Blastocystis spp.', 'Bordetella pertussis', 'Brugia malayi', 'Brugia timori', 'Bundibugyo ebolavirus <BDBV>', 'Burkholderia cepacia', 'Campylobacter spp.', 'Candida albicans', 'Candida auris', 'Candida glabrata', 'Candida krusei', 'Candida parapsilosis', 'Candida spp., other', 'Candida tropicalis', 'Capillaria hepatica', 'Capillaria philippinensis', 'Chilomastix mesnili', 'Chlamydia spp.', 'Chlamydophila pneumoniae', 'Citrobacter freundii', 'Citrobacter koseri e.g. diversus', 'Citrobacter spp., other', 'Clonorchis spp.', 'Clostridium difficile', 'Clostridium other', 'Clostridium tetani', 'Corynebacterium diphtheriae', 'Corynebacterium spp.', 'COVID-2019/ SARS-CoV2', 'Cryptosporidium spp.', 'Cryptococcus', 'Cyclospora cayetanensis', 'Cystoisospora belli', 'Cytomegalovirus <CMV>', 'Dengue', 'Dientamoeba fragilis', 'Diphyllobothrium latum', 'Dipylidium caninum', 'Dirofilaria immitis', 'Dirofilaria repens', 'Dirofilaria tenuis', 'Dracunculus medinensis', 'Echinococcosis granulosus', 'Echinococcosis multilocularis', 'Elizabethkingia meningoseptica', 'Endolimax nana', 'Encephalitozoon cuniculi', 'Encephalitozoon hellem', 'Encephalitozoon intestinalis <Septata intestinalis>', 'Enterobacter aerogenes', 'Enterobacter agglomerans', 'Enterobacter cloacae', 'Enterobacter gergoviae', 'Enterobacter sakazakii', 'Enterobacter spp., other', 'Enterobius vermicularis', 'Enterococcus faecalis', 'Enterococcus faecium', 'Enterococcus spp., other', 'Enterocytozoon bieneusi', 'Enterovirus <polio, coxsackie, echo>', 'Entamoeba coli', 'Entamoeba dispar', 'Entamoeba hartmanni', 'Entamoeba histolytica', 'Entamoeba polecki', 'Escherichia coli', 'Fasciola gigantica', 'Fasciola hepatica', 'Fasciolopsis buski', 'Filaments other', 'Flavobacterium spp.', 'Gardnerella spp.', 'Giardia lamblia / Giardia duodenalis', 'Gnathostoma spp.', 'H1N1pdm09', 'H5N1', 'H7N9', 'Haemophilus influenzae', 'Haemophilus parainfluenzae', 'Haemophilus parainfluenzae', 'Haemophilus spp., other', 'Haemophilus spp., other', 'Hafnia spp.', 'Helicobacter pylori', 'Hepatitis A virus', 'Hepatitis B virus', 'Hepatitis C virus', 'Hepatitis D virus', 'Hepatitis E virus', 'Herpes simplex virus', 'Heterophyes heterophyes', 'Human immunodeficiency virus <HIV>', 'Human papillomavirus <HPV>', 'Hymenolepis nana', 'Influenza A virus', 'Influenza B virus', 'Influenza C virus', 'Influenza D virus', 'Iodamoeba buetschlii', 'Japanese encephalitis virus <JEV>', 'Klebsiella oxytoca', 'Klebsiella pneumoniae', 'Klebsiella spp., other', 'Lactobacillus spp.', 'Legionella pneumophila serogroup', 'Legionella spp.', 'Leishmania spp.', 'Listeria monocytogenes', 'Loa loa', 'Measles morbillivirus <MeV>', 'MERS CoV', 'Monkeypox virus', 'Moraxella catharralis', 'Moraxella spp., other', 'Morganella spp.', 'Mumps orthorubulavirus', 'Mycobacterium tuberculosis complex', 'Mycobacterium, atypical', 'Mycoplasma pneumoniae', 'Mycoplasma spp.', 'Mucormycosis spp.', 'Naegleria fowleri', 'Necator americanus', 'Neisseria meningitidis', 'Neisseria spp., other', 'Nocardia spp.', 'Norovirus', 'Nosema spp.', 'Onchocerca volvulus', 'Opisthorchis felineus', 'Opisthorchis spp.', 'Opisthorchis viverrini', 'Other bacteria', 'Other coagulase-negative staphylococci <CNS>', 'Other enterobacteriaceae', 'Other fungi', 'Other Gram-negative Bacilli, non enterobacteriaceae', 'Other Gram-negative cocci', 'Other Gram-positive bacilli', 'Other Gram-positive cocci', 'Other haemol. Streptococcae <C, G>', 'Other parasite', 'Other virus', 'Paragonimus spp.', 'Parainfluenzavirus', 'Pasteurella spp.', 'Plasmodium falciparum', 'Plasmodium knowlesi', 'Plasmodium malariae', 'Plasmodium ovale', 'Plasmodium vivax', 'Pleistophora spp.', 'Pneumocystis jirovecii', 'Prevotella spp.', 'Propionibacterium spp.', 'Proteus mirabilis', 'Proteus spp., other', 'Proteus vulgaris', 'Providencia spp.', 'Pseudomonadaceae family, other', 'Pseudomonadaceae family, other', 'Pseudomonas aeruginosa', 'Pseudoterranova decipiens', 'Rabies lyssavirus', 'Respiratory syncytial virus <RSV>', 'Reston ebolavirus <RESTV>', 'Rhinovirus', 'Rotavirus', 'Rubivirus rubellae', 'Salmonella enteritidis', 'Salmonella spp., other', 'Salmonella typhi or paratyphi', 'Salmonella typhimurium', 'Sarcocystis spp.', 'Sarcoptes scabiei', 'Sarcoptes scabiei', 'SARS virus', 'Schistosoma haematobium', 'Schistosoma japonicum', 'Schistosoma mansoni', 'Serratia liquefaciens', 'Serratia marcescens', 'Serratia spp., other', 'Shigella spp.', 'Staphylococcus aureus', 'Staphylococcus epidermidis', 'Staphylococcus haemolyticus',

'Stenotrophomonas maltophilia', 'Stenotrophomonas maltophilia', 'Stenotrophomonas maltophilia', 'Streptococcus agalactiae <B>', 'Streptococcus pneumoniae', 'Streptococcus pyogenes <A>', 'Streptococcus spp., other', 'Strongyloides spp.', 'Sudan ebolavirus <SUDV>', 'Taenia asiatica', 'Taenia saginata', 'Taenia solium', 'Taenia solium', 'Taï Forest ebolavirus <TAFV>', 'Toxocara canis', 'Toxocara cati', 'Toxoplasma gondii', 'Trachipleistophora spp.', 'Trichinella spp.', 'Trichomonas tenax', 'Trichomonas vaginalis', 'Trichuris trichiura', 'Trypanosoma brucei ssp.', 'Trypanosoma cruzi', 'Tubulinosema acridophagus', 'Uncinaria stenocephala', 'Varicella-zoster virus', 'Vittaforma corneae <syn. Nosema corneum>', 'Wuchereria bancrofti', 'Yersinia spp.', 'Zaire ebolavirus <ZEBOV>', 'Zika virus']

Variable required for the calculation of quality indicators

1. VTE prophylaxis
    - Venous thromboembolism (VTE) prophylaxis
    - Ventilation
2. Target RASS
    - Target RASS score
    - Ventilation
    - Sedated
3. Actual RASS
    - Actual RASS score
    - Ventilation
    - Sedated
4. Delta RASS
    - Target RASS score
    - Actual RASS score
    - Ventilation
    - Sedated
5. Stress ulcer prophylaxis
    - Stress ulcer prophylaxis
    - Ventilation
6. Spontaneous awakening trial
    - Spontaneous awakening trial (SAT)
    - Ventilation
    - Route of ventilation
    - Sedated
7. Spontaneous breathing trial
    - Spontaneous breathing trial (SBT)
    - Ventilation
    - Route of ventilation
    - Sedated

- Lowest SpO_2
- Lowest FiO_2
- Highest respiratory rate

8. Elevated head of bed
    - Head of Bed >30° or patient
    - Ventilation
9. Mobilisation
    - Physiotherapy/ mobilisation
    - Ventilation
10. CLABSI
    Specimen report
    - Date specimen taken
    - Type of specimen
    - Type of test performed
    - Test result
    - Highest temperature
    - Cardiovascular support
    - Central venous catheter
11. CAUTI
    - Specimen report
    - Date specimen taken
    - Type of specimen
    - Type of test performed
    - Test result
    - Highest temperature
    - Urinary catheter
12. IVAC
    - Antimicrobial use
    - Antimicrobial type
    - Highest temperature
    - Highest white blood cell count
    - Lowest white blood cell count
    - Lowest FiO_2
    - Lowest PEEP
    - Ventilation
13. Pressure injury
    - Pressure sore

- Ventilation

14. Re-intubation
    - Ventilation
    - Route of ventilation
