# Supplementary material for: Contextual determinants of implementation of stakeholder-selected quality indicators in Indian intensive care units: a mixed-methods evaluation within a national registry network
Source: Front Health Serv. 2026 Jul 27;6:1872641. doi: 10.3389/frhs.2026.1872641 (PMC13454447; doi:10.3389/frhs.2026.1872641)
Supplement: Supplementary file 2 [file Datasheet2.pdf]

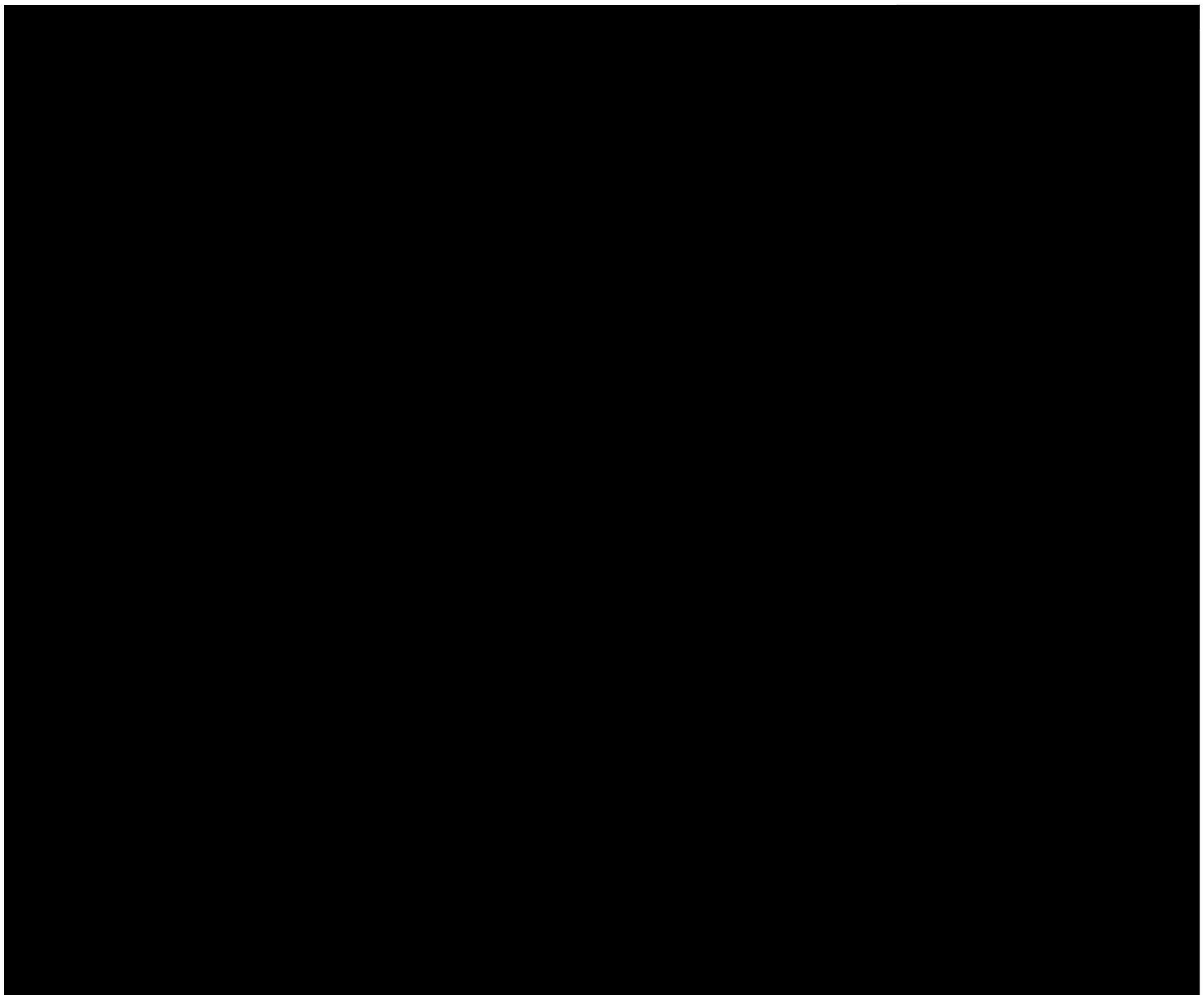

Disclaimer

This report contains confidential and clinical information and should be managed in accordance with local healthcare data regulations.

Contents

Disclaimer . . . 1
Data Quality . . . 3
Case mix, severity of illness, and outcomes . . . 5
Critical care encounters . . . 6
ICU Mortality . . . 8
Indicators of quality . . . 10

List of Figures

1 Monthly Number of Intensive Care Units (ICUs) participating in the registry . . . 4
2 Number of completed encounters submitted to the registry . . . 6
3 Observed and APACHE II predicted mortality by ventilation status . . . 8
4 Observed and predicted mortality for the last 12 months (calculated using APACHE II) . . . 8
5 Standardised Mortality Ratios for the reporting period, with APACHE II and eTropICS . . . 9
6 VTE prophylaxis in mechanically ventilated patients . . . 11
7 Target RASS rates . . . 12
8 Actual RASS rates . . . 12
9 Delta RASS . . . 13
10 Stress ulcer prophylaxis rates . . . 13
11 Spontaneous awakening trial (SAT) rates . . . 14
12 Spontaneous breathing trial (SBT) rates . . . 14
13 Spontaneous breathing trial (SBT) conducted in eligible and non eligible patients . 15
14 Head of bed elevation in mechanically ventilated patients . . . 15
15 Mobilisation in mechanically ventilated patients . . . 16
16 Culture obtained within 24 hours of new antibiotic commencement . . . 16
17 Incidence of CLASBI for patients with a CVC insitu for >= 2 consecutive days . . 17
18 Incidence of CAUTI for patients who ever had a urinary catheter . . . 17
19 Incidence of IVAC for patients who were mechanically ventilated for >= 2 consecutive days . . . 18
20 Pressure injury FREE rates . . . 18
21 Re-intubation rates . . . 19
22 After hours discharge and unplanned readmission to ICU within 48 hours . . . 19

List of Tables

1 Data Quality in this ICU vs all ICUs . . . 3
2 Characteristics of patients discharged from adult ICUs between 01 January 2024 and 31 January 2024 . . . 5

3 Top 10 APACHE IV reasons for ICU admission all ICUs between 2024-01-01 and 2024-01-31 6
4 Top 10 APACHE IV reasons for ICU admission this ICU between 2024-01-01 and 2024-01-31 7
5 Incidence of notifiable diseases (identified from SNOMED diagnosis entries) in all ICUs between 2024-01-01 and 2024-01-31 7
6 Incidence of notifiable diseases (identified from SNOMED diagnosis entries) in this ICU between 2024-01-01 and 2024-01-31 7
7 Indicators of quality between 01 January 2024 and 31 January 2024 10

Data Quality

The importance of Registry data as a source for service evaluation, benchmarking, quality improvement and research continues to grow. Central to the value of registry data to these activities is its quality. Data quality is determined by several factors; completeness, validity, consistency, reliability and timeliness. Completeness; Over the year the registry captured data on more than 83% of all ICU admissions to participating units. Completeness of data is assessed via routine validation of data (minimum weekly) in ICUs. Validity; data contributing to observational and interventional research is subject to source data verification. Consistency and reliability; Definitions, data collection guidance, training of data collectors and ongoing feedback of data through reports, near real dashboards and access to de-identified data promotes data quality.

Table 1: Data Quality in this ICU vs all ICUs

Month	Type	% of required validation censuses completed	% of admitted patients reported in platform Median - (IQR)	% of admissions with all Daily Q forms filled in
**2023-02**	This ICU	-	-	65.26
	All ICUs	-	-	29.92
**2023-03**	This ICU	-	-	62.41
	All ICUs	-	-	33.7
**2023-04**	This ICU	-	-	53.15
	All ICUs	-	-	42.17
**2023-05**	This ICU	-	-	59.17
	All ICUs	-	-	44.9
**2023-06**	This ICU	-	-	51.85
	All ICUs	-	-	42.35
**2023-07**	This ICU	-	-	57.65
	All ICUs	-	-	44.48
**2023-08**	This ICU	-	-	50.44
	All ICUs	8.28	72.3 (28.1 - 100)	45.53
**2023-09**	This ICU	70	102.7	50.46
	All ICUs	59.11	92.9 (63.1 - 100)	44.79
**2023-10**	This ICU	45.16	98.1	51.52
	All ICUs	76.02	97.6 (83 - 100)	47.94
**2023-11**	This ICU	93.33	100	60.22
	All ICUs	60.67	100 (96.3 - 100)	45.99
**2023-12**	This ICU	-	-	37.89
	All ICUs	47.42	99.4 (93.3 - 100)	52.51
**2024-01**	This ICU	-	-	54.12
	All ICUs	59.46	100 (89.4 - 100)	49.87

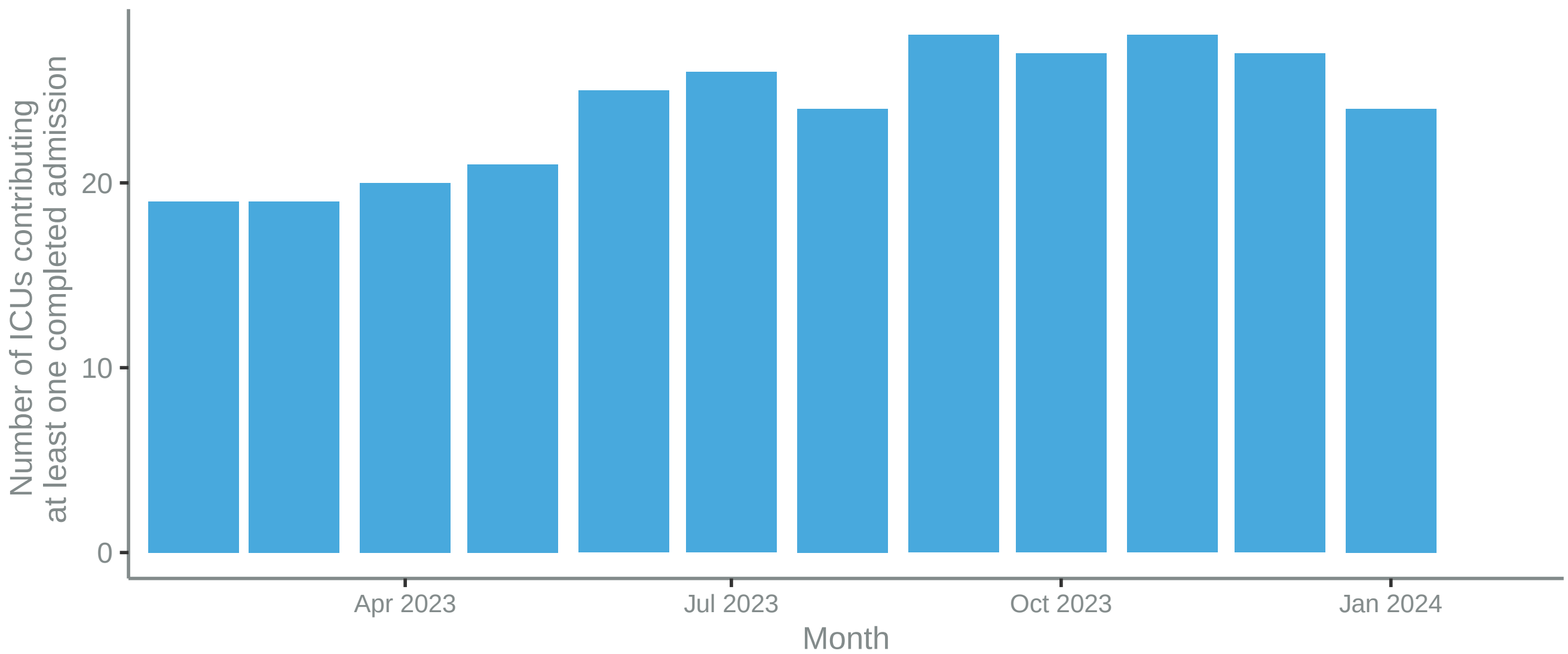

Figure 1: Monthly Number of Intensive Care Units (ICUs) participating in the registry

Case mix, severity of illness, and outcomes

Note: The remaining sections of the report include the patients discharged during the observation period. Patients admitted, but not discharged, are excluded.

Table 2: Characteristics of patients discharged from adult ICUs between 01 January 2024 and 31 January 2024

Variable	All ICU	This ICU
**Patient Characteristics**		
Sites N	24	
Patients discharged N	1813	90
Age (years) Median (IQR)	60 (45 - 71)	66 (53 - 74)
Male N(%)	1098 (60.56)	49 (54.44)
Planned admissions after elective surgery N(%)	253 (13.95)	4 (4.44)
**Source of Admission**		
Ward	363 (20.02)	21 (23.33)
Home	0 (0)	0 (0)
Operating theatre	341 (18.81)	3 (3.33)
Emergency department	1030 (56.81)	61 (67.78)
ICU/HDU	59 (3.25)	5 (5.56)
Missing	20 (1.1)	0 (0)
**Organ support at admission**		
Mechanical ventilatory support [1] N(%)	479 (26.42)	35 (38.89)
Invasive mechanical ventilation [2] N(%)	326 (17.98)	20 (22.22)
NIV N(%)	153 (8.44)	15 (16.67)
Vasoactives N(%)	271 (14.95)	27 (30)
Tracheostomy N(%)	27 (1.49)	0 (0)
Renal replacement therapy N(%)	54 (2.98)	6 (6.67)
**Organ support during ICU stay [3]**		
Mechanical ventilatory support N(%)	553 (33.17)	45 (50)
Invasive mechanical ventilation N(%)	403 (24.18)	31 (34.44)
NIV N(%)	193 (11.58)	18 (20)
Tracheostomy N(%)	58 (3.48)	6 (6.67)
Renal replacement therapy N(%)	93 (5.58)	6 (6.67)
**Severity of Illness**		
APACHE II score Median (IQR)	10 (6 - 16)	10 (7 - 19)
APACHE II Predicted risk of death Median (IQR)	0.08 (0.04 - 0.17)	0.1 (0.06 - 0.28)
eTropICS Predicted risk of death Median (IQR)	0.17 (0.12 - 0.41)	0.29 (0.17 - 0.59)
**Outcomes**		
ICU Mortality N(%)	163 (8.99)	5 (5.56)
Hospital Mortality N(%)	177 (9.76)	6 (6.67)
eTropICS SMR Median (IQR)	0.42 (0.17 - 0.63)	0.19
APACHE II SMR Median (IQR)	0.87 (0.48 - 1.48)	0.53
Survivors discharged home from ICU N(%)	227 (13.76)	28 (32.94)
Survivors transferred to another facility from ICU N(%)	43 (2.61)	0 (0)

[1] Mechanical ventilation includes both invasive and non-invasive vantilation
[2] Invasive ventilation includes tracheostomy
[3] It's only reported if the site has daily data

Critical care encounters

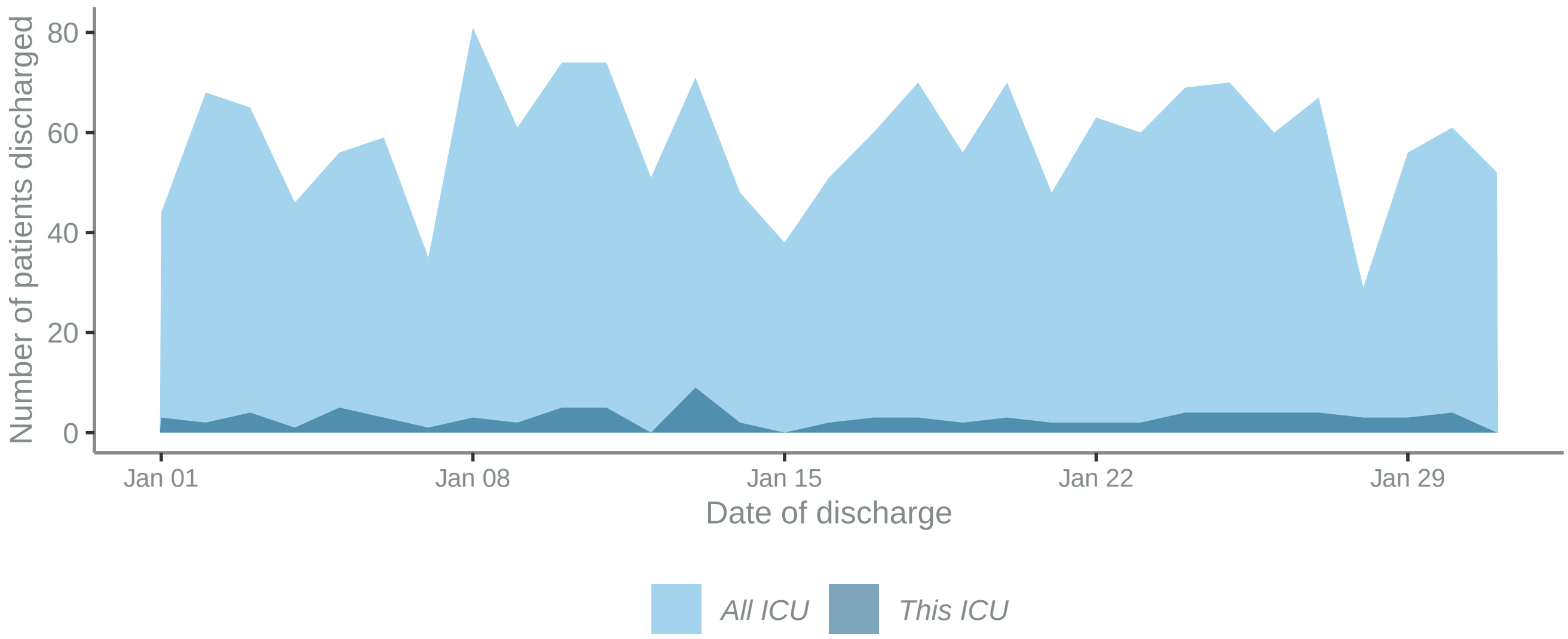

Figure 2: Number of completed encounters submitted to the registry

Table 3: Top 10 APACHE IV reasons for ICU admission all ICUs between 2024-01-01 and 2024-01-31

Diagnosis	Count	% of admissions
Pneumonia, other	152	8.4
Sepsis, other	138	7.63
Genitourinary medical, other	135	7.46
Infarction, acute myocardial (MI), none of the above	130	7.19
Cardiovascular medical, other	114	6.3
Respiratory- medical, other	109	6.03
CVA, cerebrovascular accident/stroke	104	5.75
Metabolic/endocrine medical, other	99	5.47
GI medical, other	84	4.64
Neurologic medical, other	71	3.92

Table 4: Top 10 APACHE IV reasons for ICU admission this ICU between 2024-01-01 and 2024-01-31

Diagnosis	Count	% of admissions
Pneumonia, other	18	20
Sepsis, other	15	16.67
Sepsis, renal/UTI (including bladder)	12	13.33
Cardiovascular medical, other	10	11.11
Respiratory- medical, other	10	11.11
CHF, congestive heart failure	9	10
Infarction, acute myocardial (MI), none of the above	8	8.89
GI medical, other	7	7.78
Emphysema/bronchitis	6	6.67
ARDS-adult respiratory distress syndrome, non-cardiogenic pulmonary edema	5	5.56

Table 5: Incidence of notifiable diseases (identified from SNOMED diagnosis entries) in all ICUs between 2024-01-01 and 2024-01-31

Disease	Count	% of admissions
Dengue	16	0.88
Acute meningitis	11	0.61
Acute encephalitis	4	0.22
Acute infectious hepatitis	4	0.22
ARI/Influenza Like Illness(ILI)	4	0.22
Jaundice of < 4 weeks	3	0.17
Animal Bite - Snake Bite	1	0.06
Enteric fever (typhoid or paratyphoid fever)	1	0.06
Measles	1	0.06
Scrub Typhus	1	0.06

Note:
Notifiable diseases were taken from here:
https://idsp.mohfw.gov.in/index1.php?lang=1&level=1&sublinkid=5985&lid=3925

Table 6: Incidence of notifiable diseases (identified from SNOMED diagnosis entries) in this ICU between 2024-01-01 and 2024-01-31

Disease	Count	% of admissions
Acute encephalitis	1	1.11
Acute infectious hepatitis	1	1.11
Acute meningitis	1	1.11
ARI/Influenza Like Illness(ILI)	1	1.11
Scrub Typhus	1	1.11

Note:
Notifiable diseases were taken from here:
https://idsp.mohfw.gov.in/index1.php?lang=1&level=1&sublinkid=5985&lid=3925

ICU Mortality

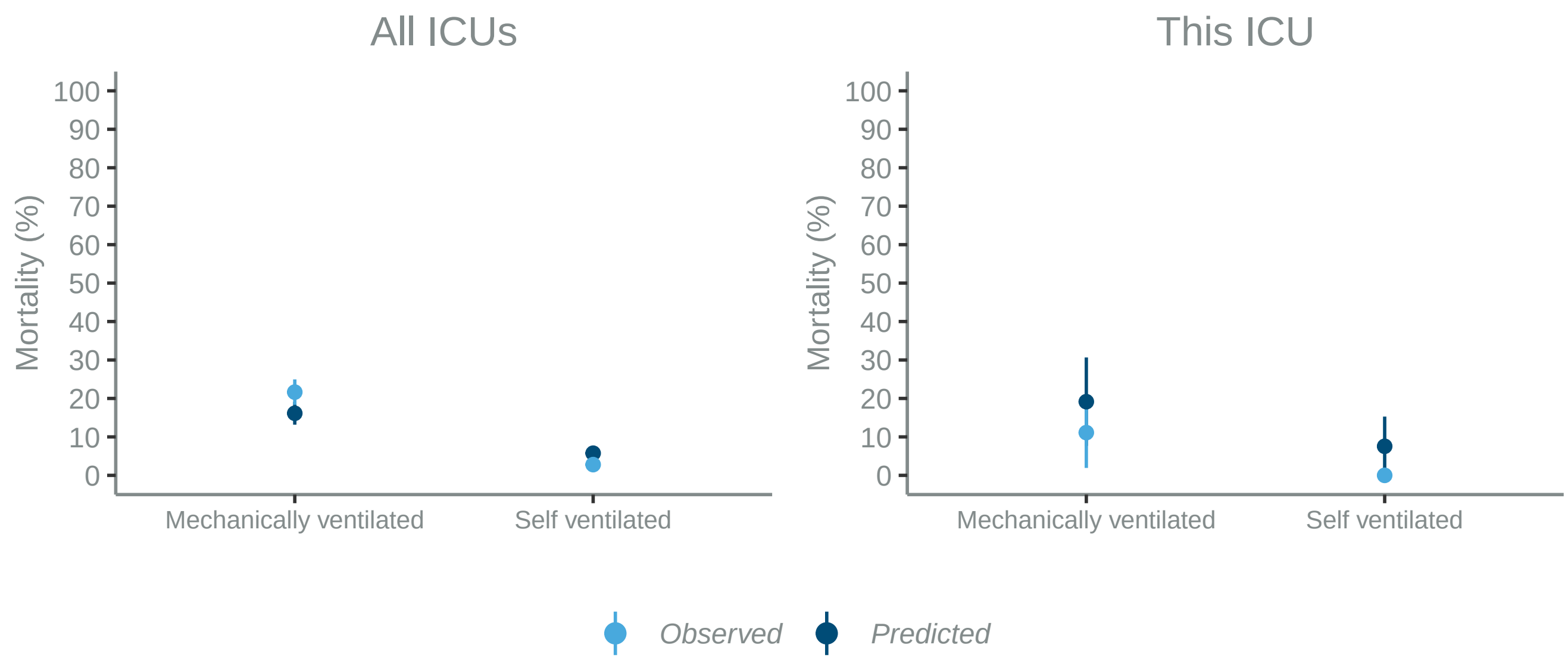

Figure 3: Observed and APACHE II predicted mortality by ventilation status
Ventilation is defined over the ICU stay. Mechanical ventilation includes NIV.

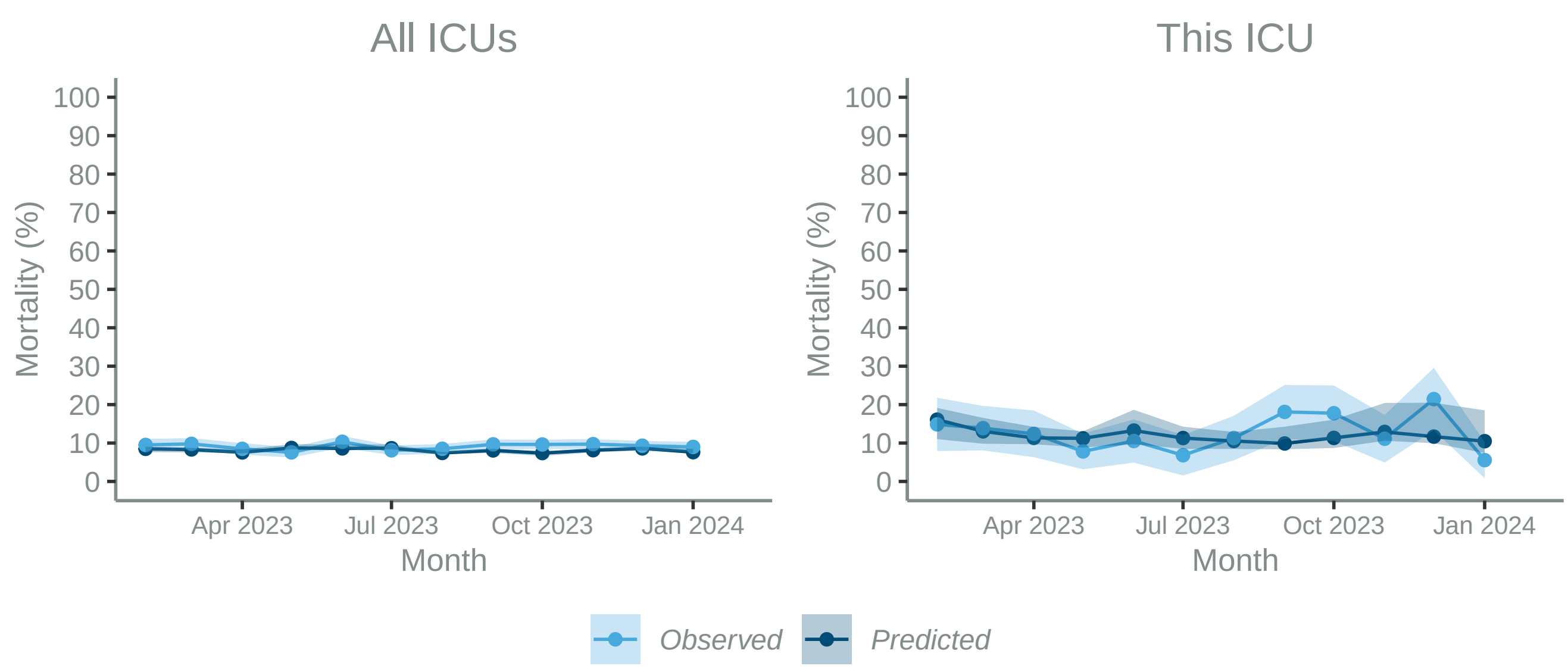

Figure 4: Observed and predicted mortality for the last 12 months (calculated using APACHE II)

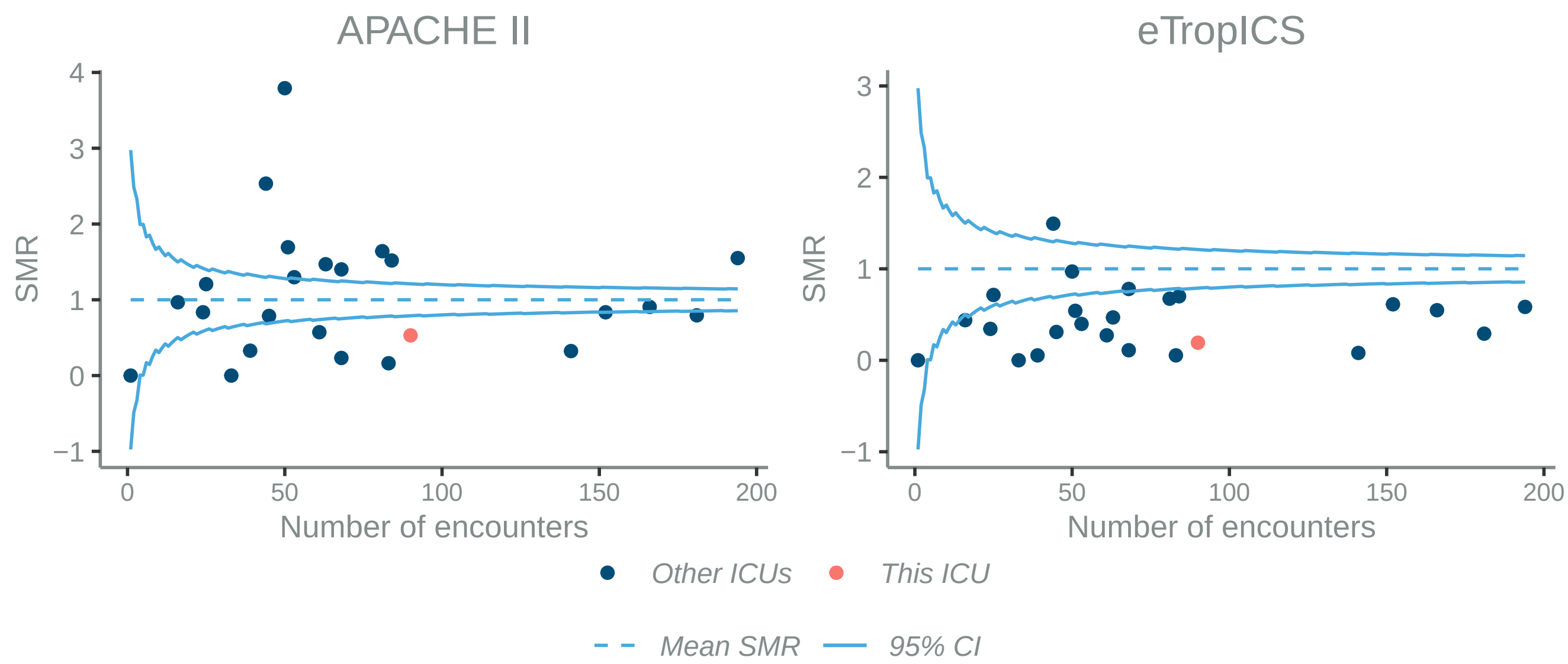

Figure 5: Standardised Mortality Ratios for the reporting period, with APACHE II and eTropICS
An SMR > 1 indicates that ICU mortality was higher than expected, while an SMR < 1 indicates ICU mortality was lower than expected

Indicators of quality

Note: For the indicators of quality, these are reported as either per eligible patient, or per eligible patient days. If information on eligibility for a given patient or patient day is missing, then it is excluded from the denominator. If information on the treatment, intervention or event of interest (numerator), is missing, then it is considered as not given, or no event. Missing information to determine either eligibility and treatment or event occurrence may result in under reporting.

Table 7: Indicators of quality between 01 January 2024 and 31 January 2024

Variables	All ICU	This ICU
Patients discharged N	1813	90
**Care processes**		
Days of VTE prophylaxis [1] N(%)	1025 (90.5)	180 (84.5)
Duration of mechanical ventilation (days) Median (IQR)	2 (1 - 3)	3 (2 - 8)
Duration of invasive ventilation (days) Median (IQR)	2 (1 - 3.75)	5 (3 - 9.5)
Duration of non-invasive ventilation (days) Median (IQR)	1 (1 - 2)	1 (1 - 2)
Duration of central venous catheter (days) Median (IQR)	2 (1 - 5)	-
Days with target RASS set [1] N(%)	120 (80.5)	-
Days with actual RASS score recorded [1] N(%)	124 (83.2)	-
Delta RASS (Target RASS - Actual RASS) Median (IQR)	2 (2 - 3)	-
Days of stress ulcer prophylaxis [1] N(%)	1064 (86.7)	69 (32.4)
Days with SAT [1] N(%)	37 (25)	-
Days with SBT [1] N(%)	218 (44.3)	-
Eligible days with SBT performed [2] N(%)	218 (63.7)	0 (0)
Days with elevated head of bed [1] N(%)	642 (52.3)	0 (0)
Days of mobilisation [1] N(%)	526 (43.5)	0 (0)
Duration of antibiotics (days) Median (IQR)	2 (1 - 4)	4 (3 - 8)
Culture obtained within 24 hours of antibiotic commencement N(%)	180 (17.91)	0 (0)
**Quality impacts**		
ICU Length of stay (days) Median (IQR)	3 (2 - 5)	5 (3 - 9)
Hospital Length of stay (days) Median (IQR)	8 (5 - 13)	9 (7 - 16)
CLABSI [3] N(%)	0 (0)	-
CLABSI per 1000 CVC days	0	-
CVC duration when CLABSI suspected (days) Median (IQR)	-	-
CAUTI [4] N(%)	1 (0.15)	-
CAUTI per 1000 urinary catheter days	0.44	-
Urinary catheter duration when CAUTI suspected (days) Median (IQR)	1 (1 - 1)	-
IVAC [5] N(%)	1 (0.5)	0 (0)
IVAC per 1000 ventilator days	0.59	0
New pressure injury [5] N(%)	17 (2.85)	8 (17.78)
Discharge after hours (between 7pm and 7am) N(%)	554 (30.56)	52 (57.78)
Unplanned readmission to ICU within 48 hours N(%)	33 (1.82)	0 (0)
Re-Intubation [6] N(%)	14 (7.53)	1 (7.14)

[1] Denominator is number of eligible ICU days
[2] Denominator is number of SBTs performed
[3] For patients with central venous catheters only
[4] For patients with urinary catheters only
[5] For mechanically ventilated patients only
[6] For extubated patients only

Note 1: If an ICU doesn't collect a particular QI, please ignore the graph for that QI

Note 2: For the following stacked bar graphs, the percentages represented by the dark blue sections are printed in grey above each bar.

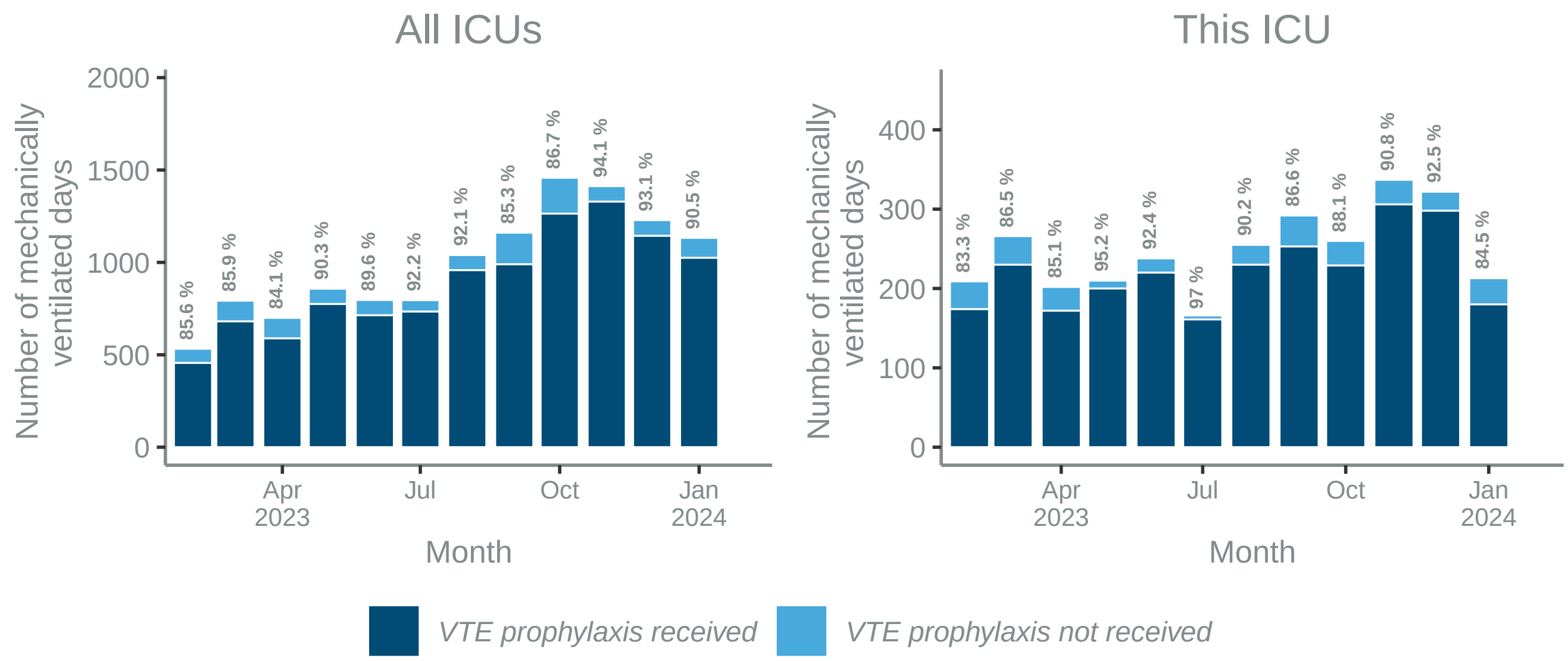

Figure 6: VTE prophylaxis in mechanically ventilated patients
Proportion of patient days for mechanically ventilated patients, where patients received VTE prophylaxis with no contraindication, was reported.

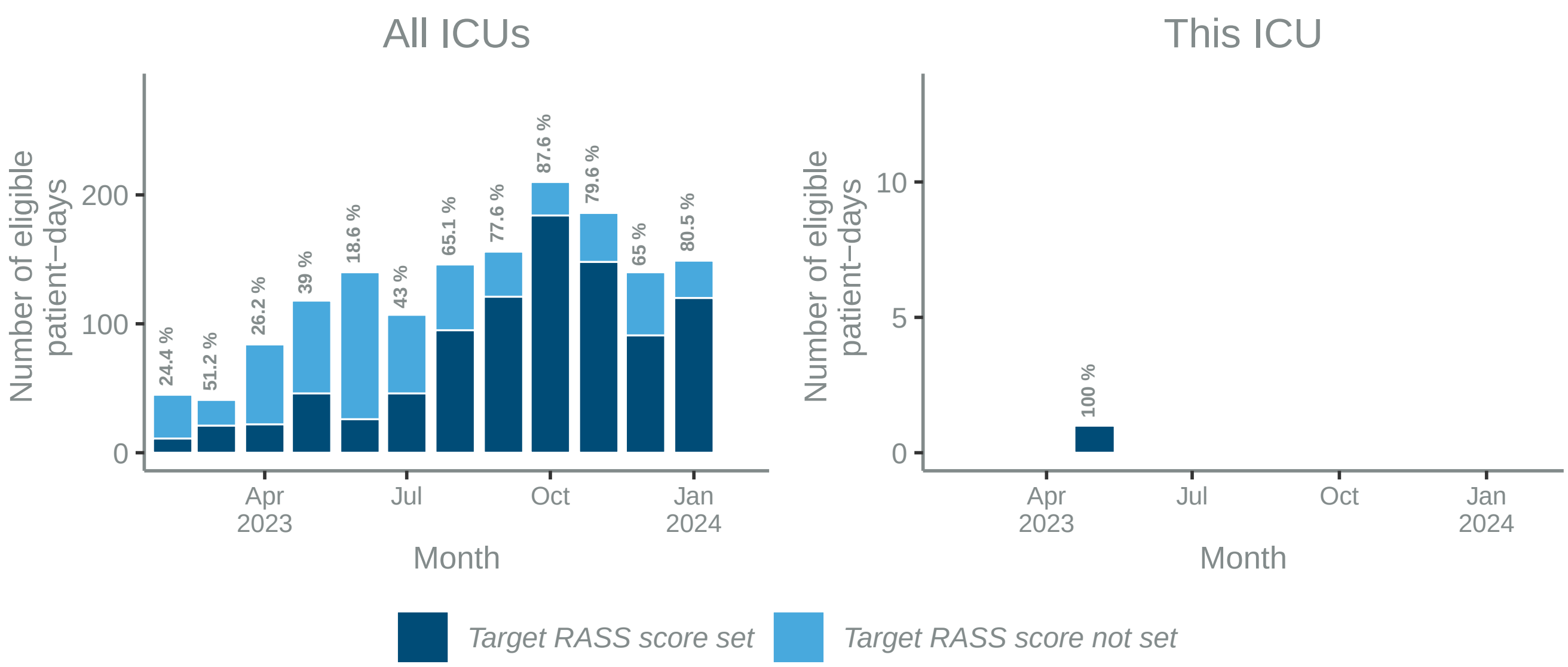

Figure 7: Target RASS rates
The patient days where a target RASS was set, are reported as a percentage of total patient days for eligible patients. Patients are eligible if they are both mechanically ventilated and sedated on a day.

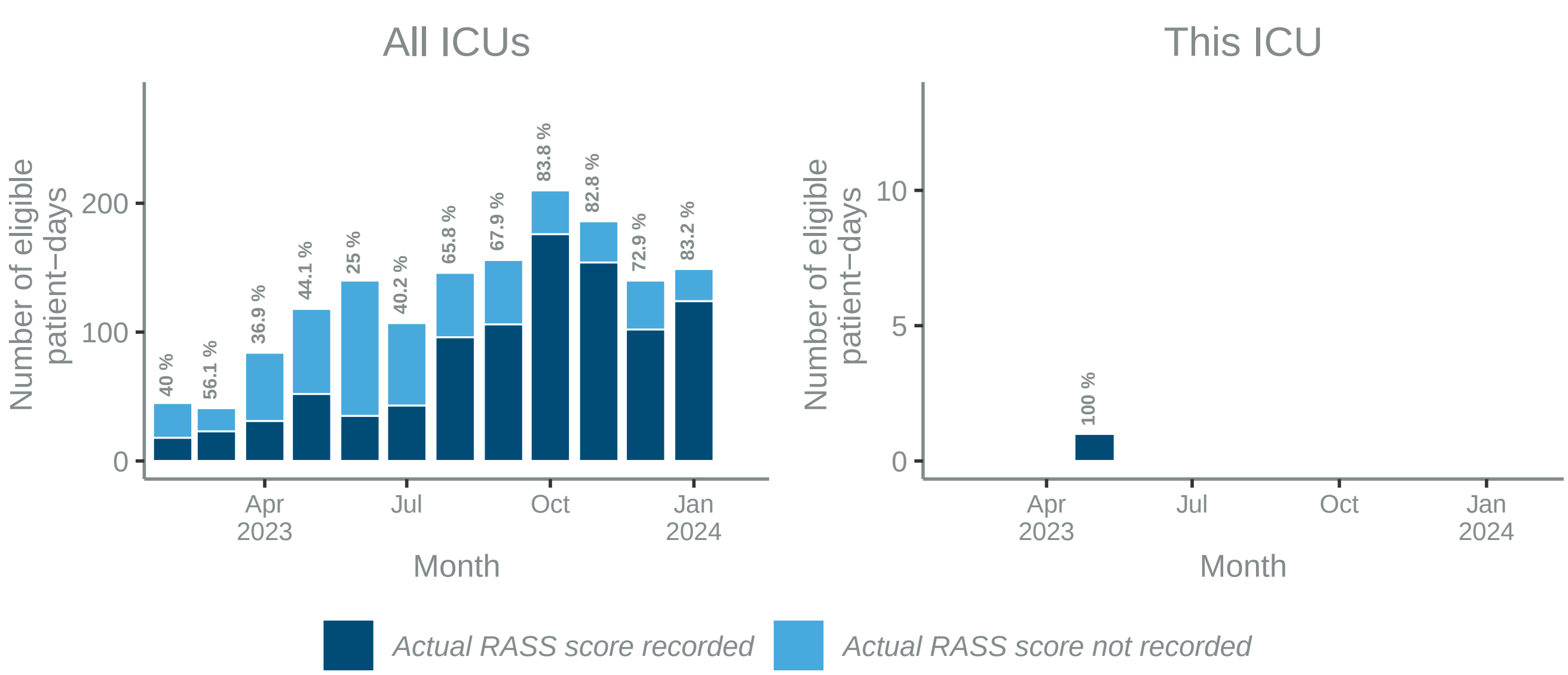

Figure 8: Actual RASS rates
The patient days where an actual RASS was recorded, are reported as a percentage of total patient days for eligible patients. Patients are eligible if they are both mechanically ventilated and sedated on a day.

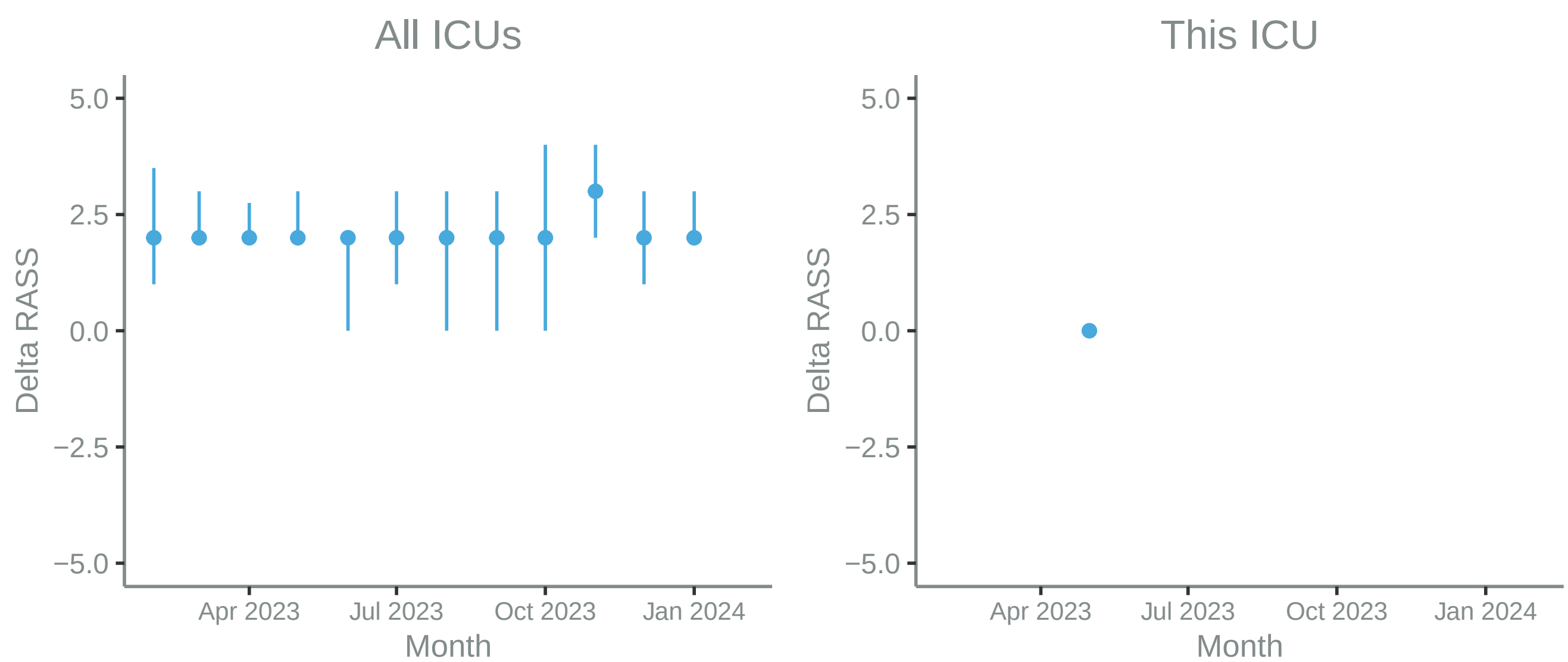

Figure 9: Delta RASS
The point displays the median of delta RASS and the edges of lines display the 1st and 3rd quartile. DELTA RASS is the difference between the target RASS and the actual RASS scores. If the Delta RASS is positive, then the target RASS score was higher than the actual RASS score. An example may be if the target score was +1, but the actual score was -2. If the Delta RASS is negative, the actual RASS score was higher than the target.

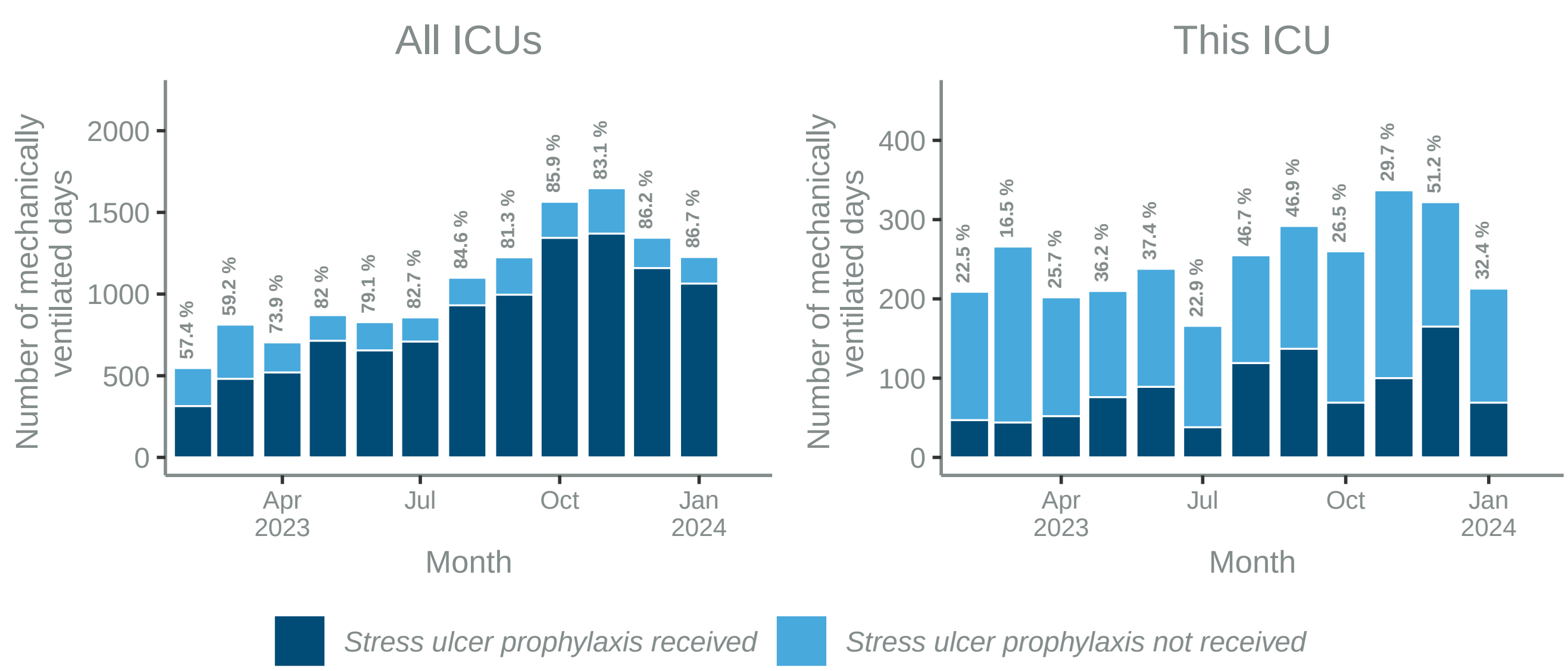

Figure 10: Stress ulcer prophylaxis rates
Proportion of patient days for mechanically ventilated patients, where patients received stress ulcer prophylaxis.

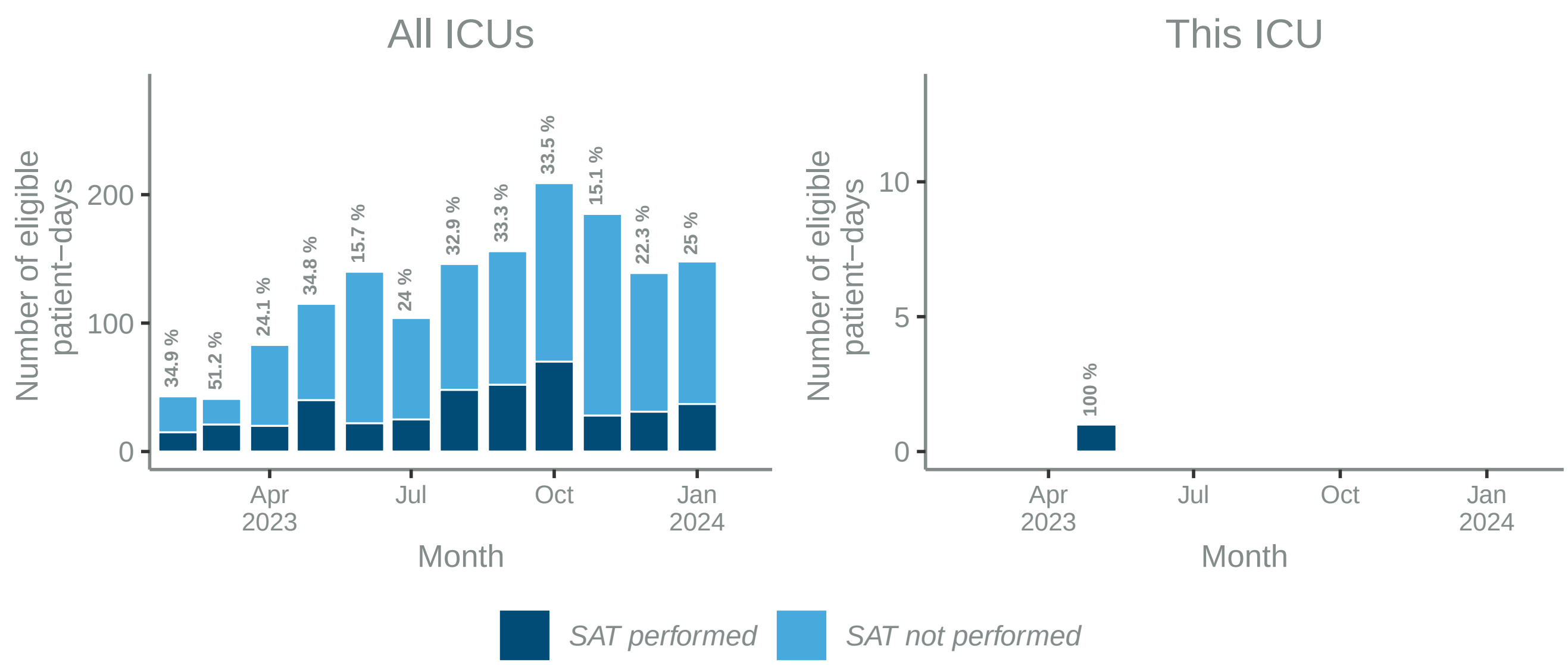

Figure 11: Spontaneous awakening trial (SAT) rates
The patient days where a SAT is performed, is reported as a percentage of total patient days for eligible patients. Patients are eligible for SAT if they are invasively ventilated and sedated.

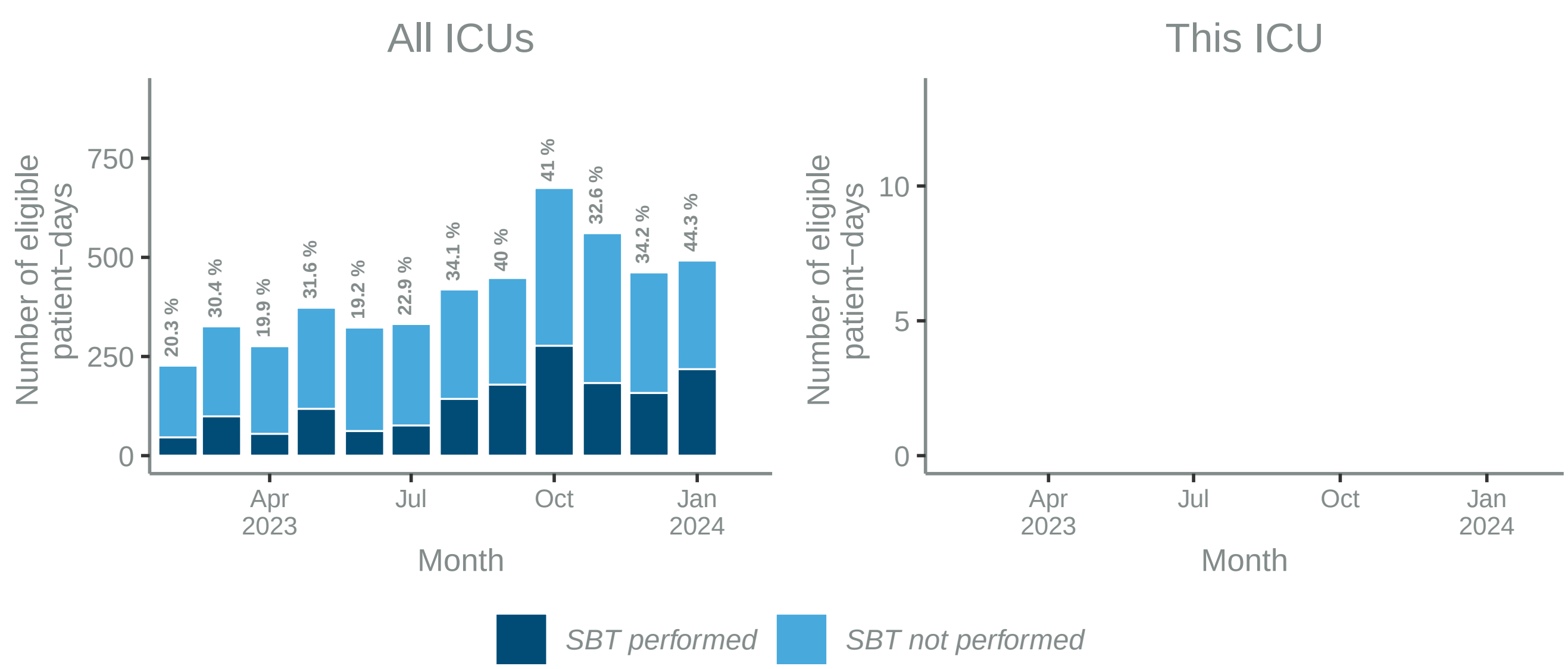

Figure 12: Spontaneous breathing trial (SBT) rates
The patient days where a SBT is performed, is reported as a percentage of total patient days for eligible patients. Patients are eligible for SBT if they are invasively ventilated, SpO2 >=88, FiO2 <= 0.5 and respiratory rate <= 35.

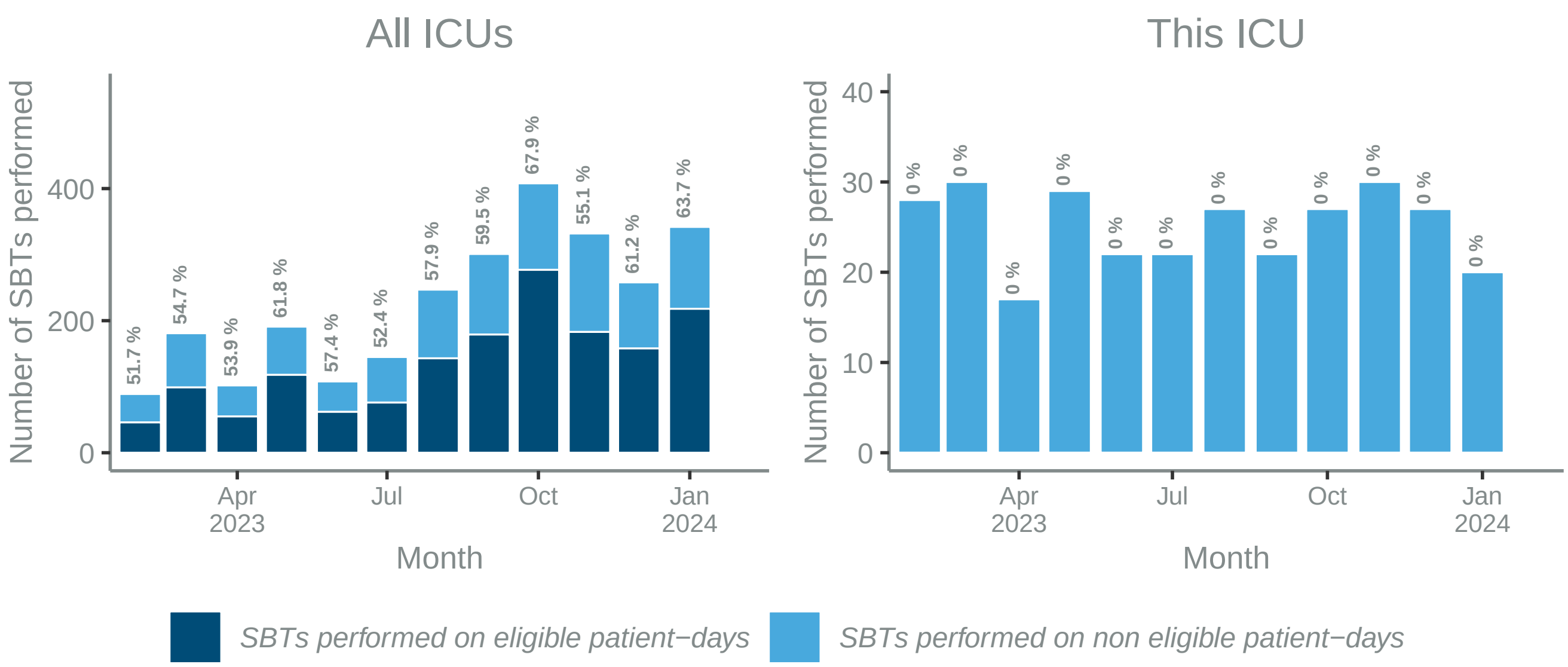

Figure 13: Spontaneous breathing trial (SBT) conducted in eligible and non eligible patients
The proportion of SBTs performed whereby the patient met criteria for SBT; invasively ventilated, SpO2 >=88, FiO2 <= 0.5 and respiratory rate <= 35.

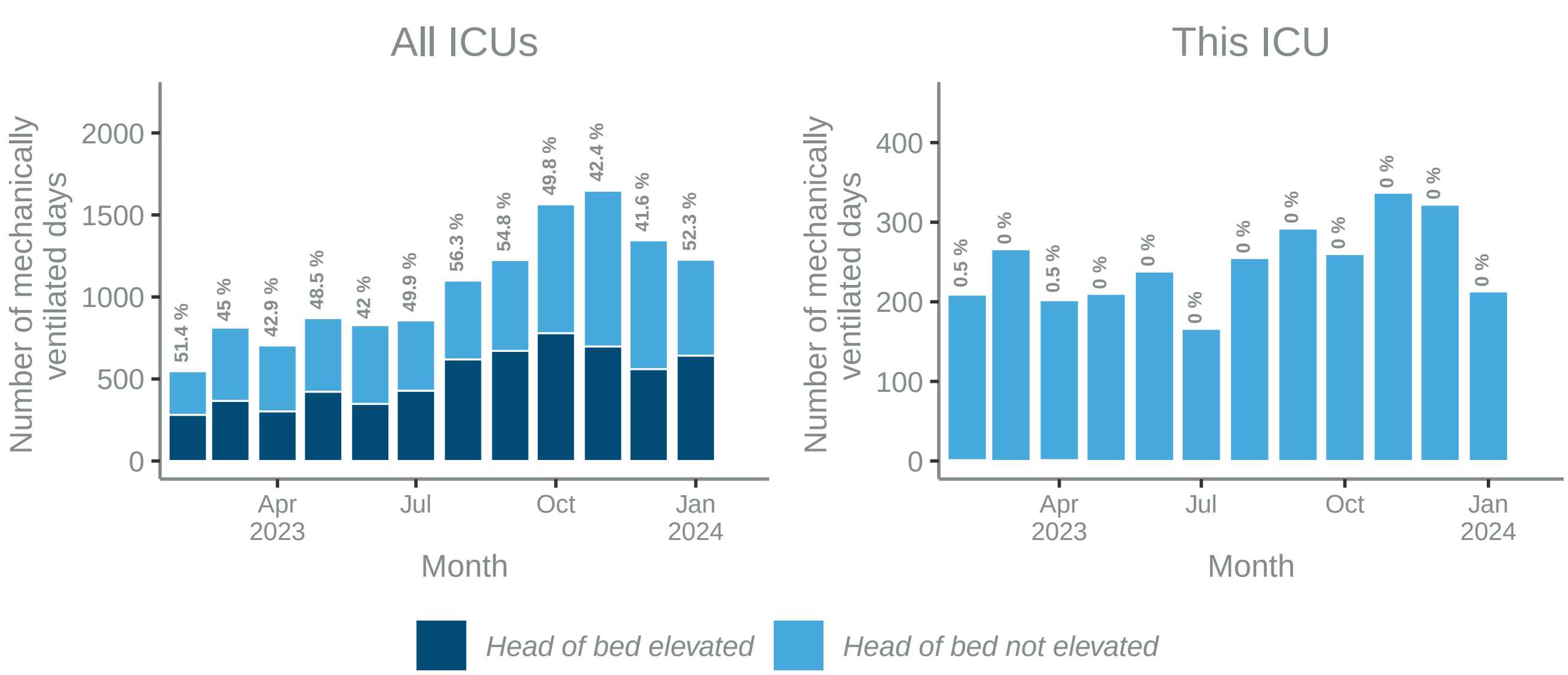

Figure 14: Head of bed elevation in mechanically ventilated patients
Compliance of head of bed elevation as a proportion of total patient days for mechanically ventilated patients.

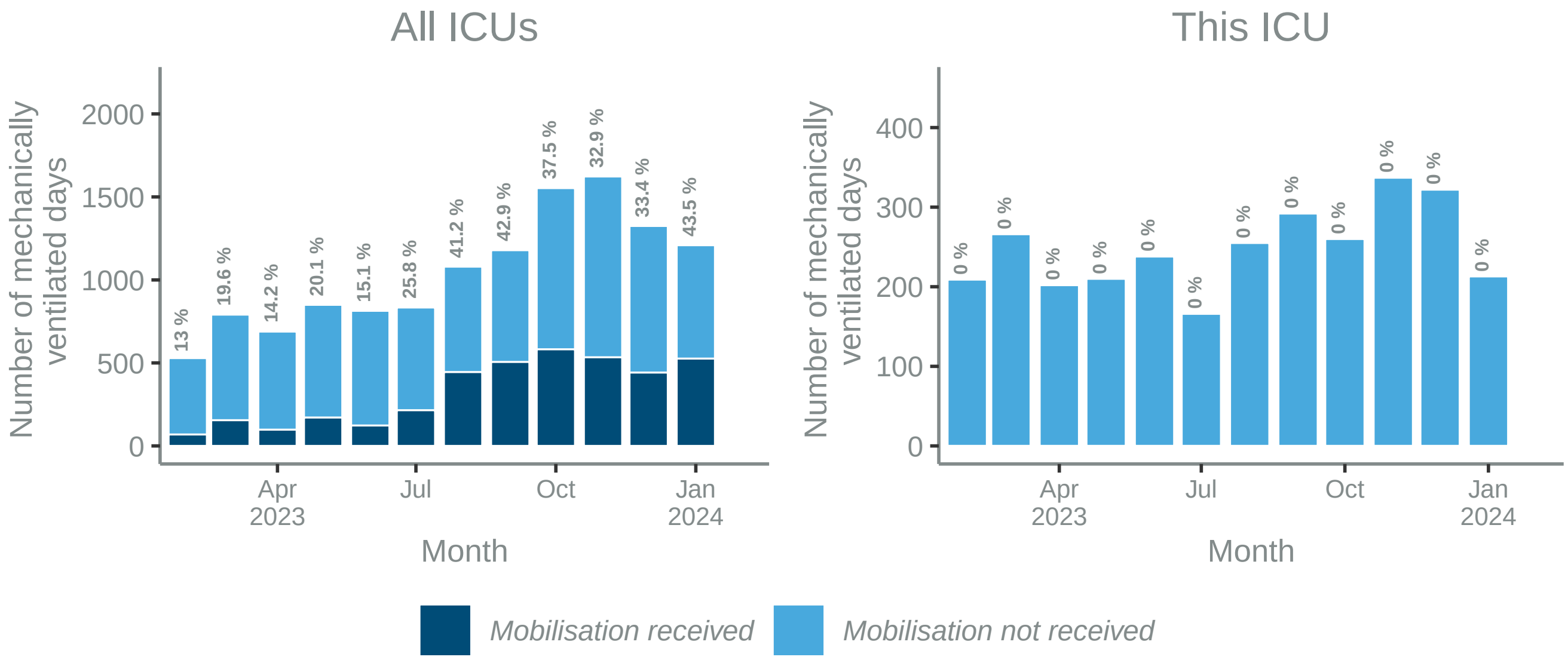

Figure 15: Mobilisation in mechanically ventilated patients
Proportion of patient days for mechanically ventilated patients, where patients were mobilised, and where no contraindication was reported.

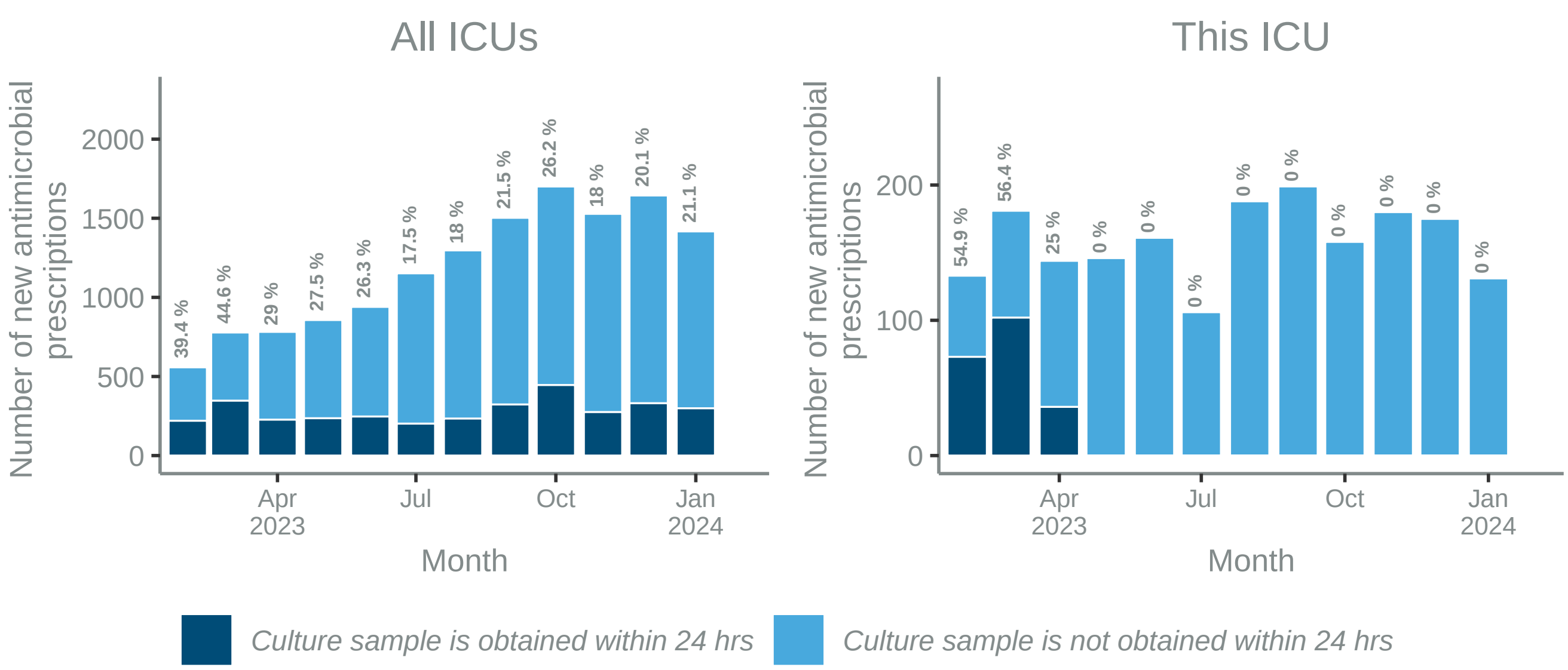

Figure 16: Culture obtained within 24 hours of new antibiotic commencement
Proportion of new antimicrobial prescriptions whereby a culture sample is obtained within 24 hrs.

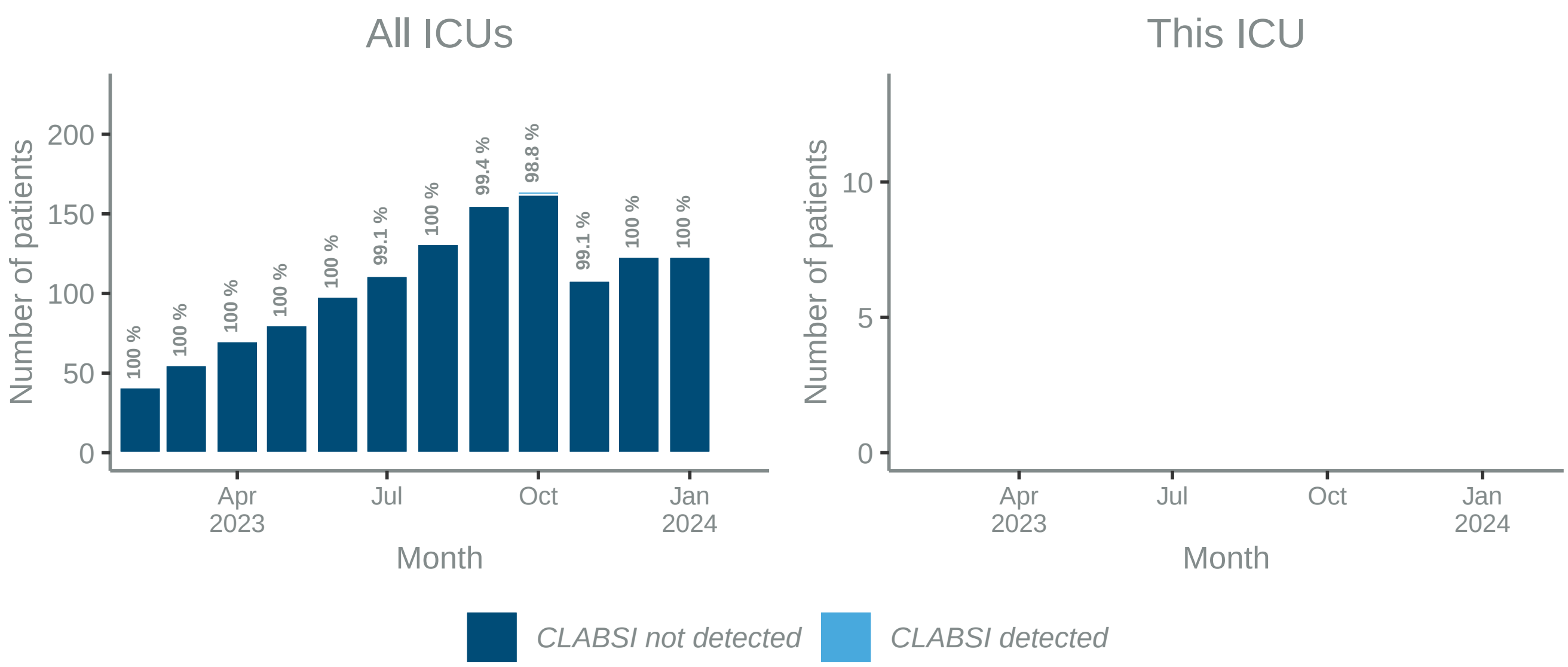

Figure 17: Incidence of CLASBI for patients with a CVC insitu for >= 2 consecutive days
CLABSI is identified as CVC insitu>= 2 consecutive days, and (fever or hypotension), and positive blood culture.

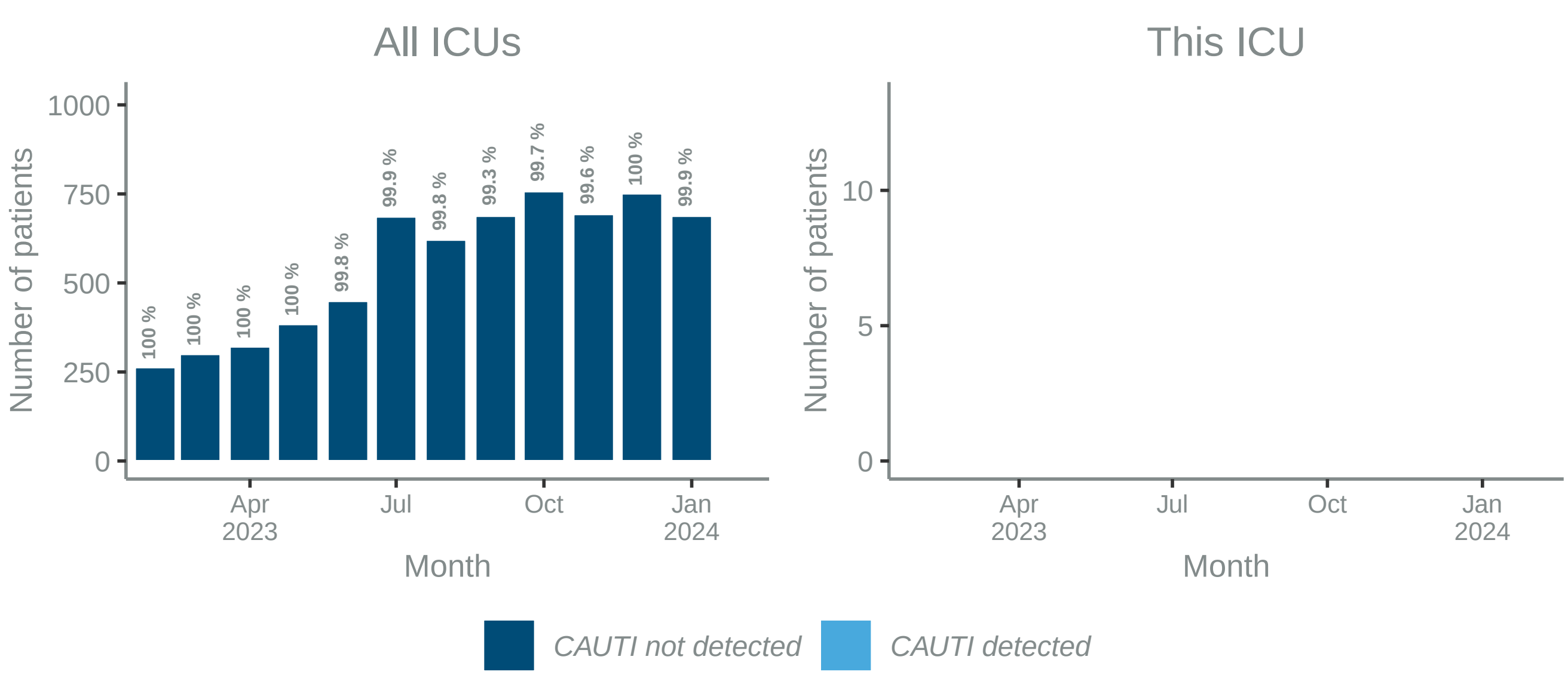

Figure 18: Incidence of CAUTI for patients who ever had a urinary catheter
CAUTI is identified as positive urine culture and fever on the same day or any day after urinary catheter was insitu.

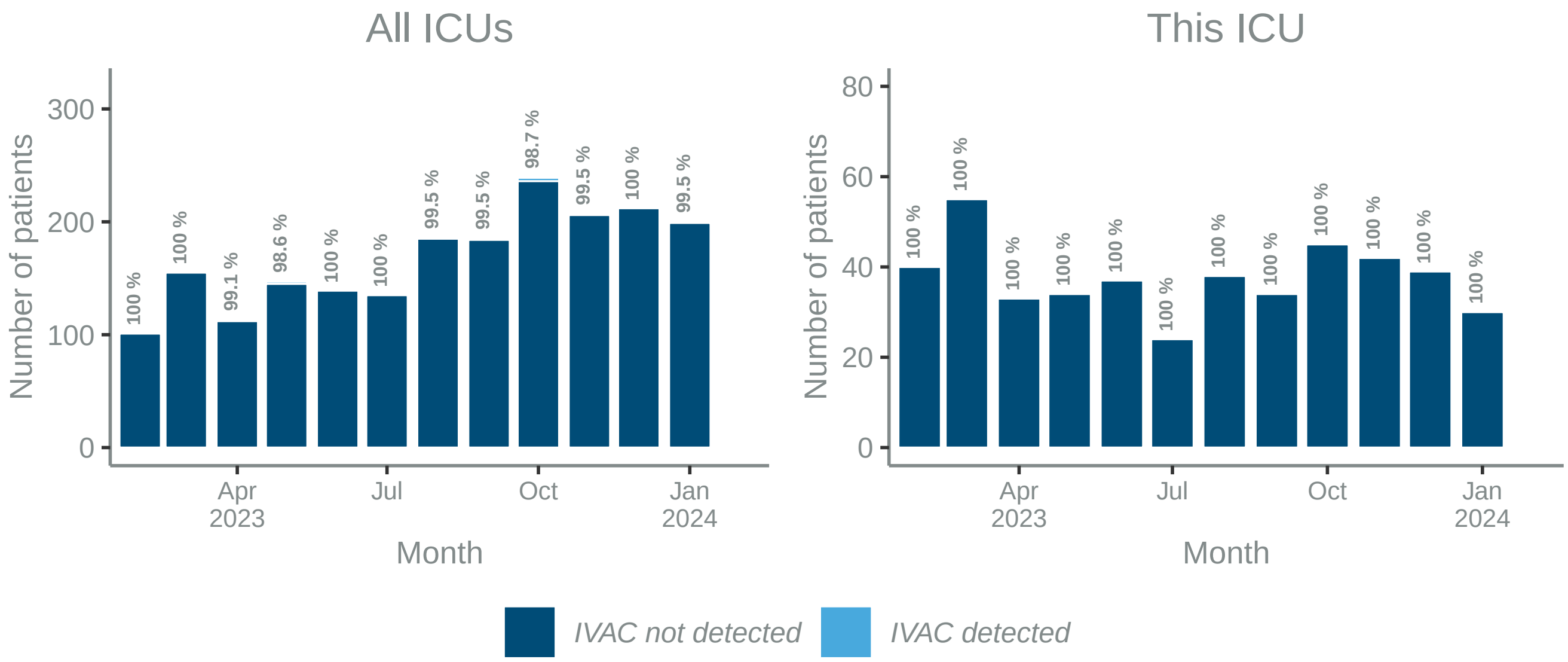

Figure 19: Incidence of IVAC for patients who were mechanically ventilated for >= 2 consecutive days IVAC is identified as mechanically ventilated for >= 2 consecutive days, and new antibiotic commencement, and (temperature > 38 degree celcius or < 36 degree celcius or WBC >=12000 cells per mm3 or <=4000 cells per mm3), and (increase in FiO2 of >=0.20 over the previous day reported value or increase in PEEP value of >=3 cmH2O over the previous day reported value).

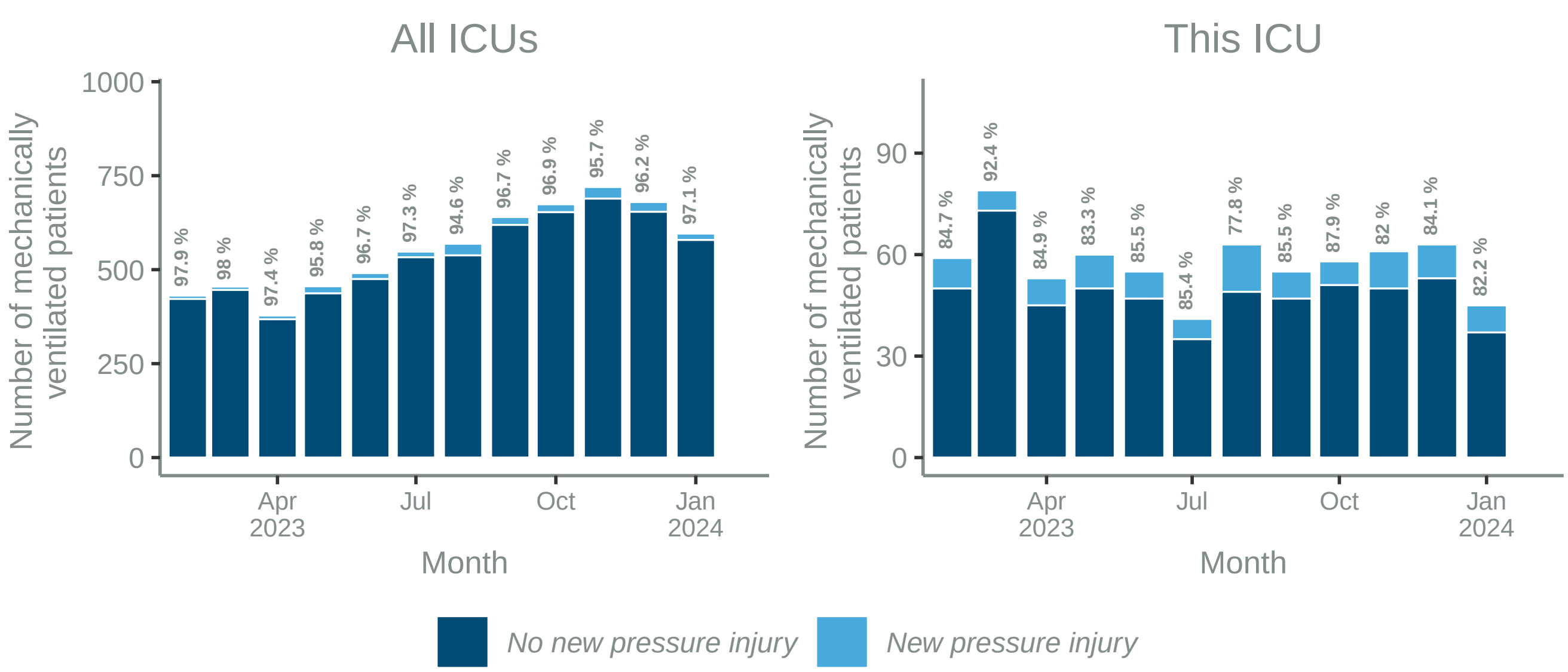

Figure 20: Pressure injury FREE rates
Proportion of mechanically ventilated patients where no new pressure injury was identified.

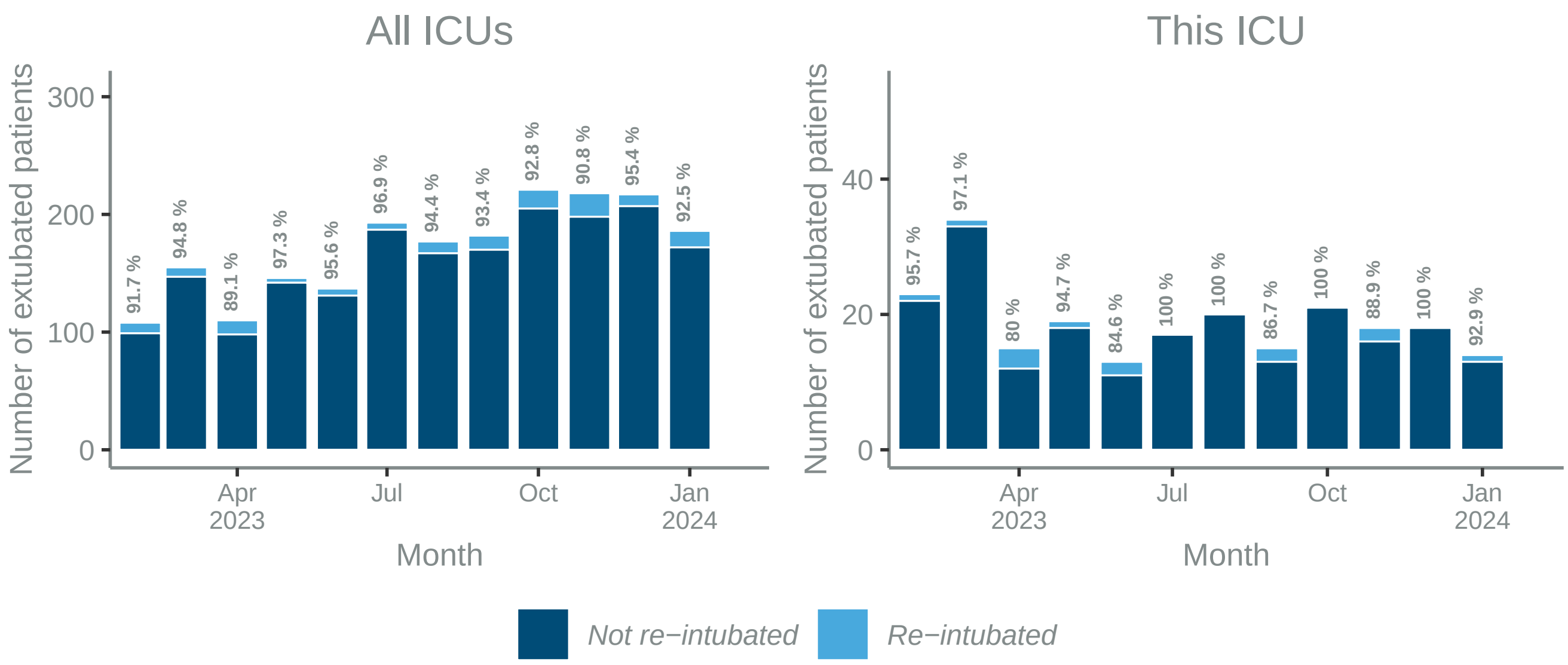

Figure 21: Re-intubation rates
Proportion of patients reintubated within 24hrs during the same ICU encounter.

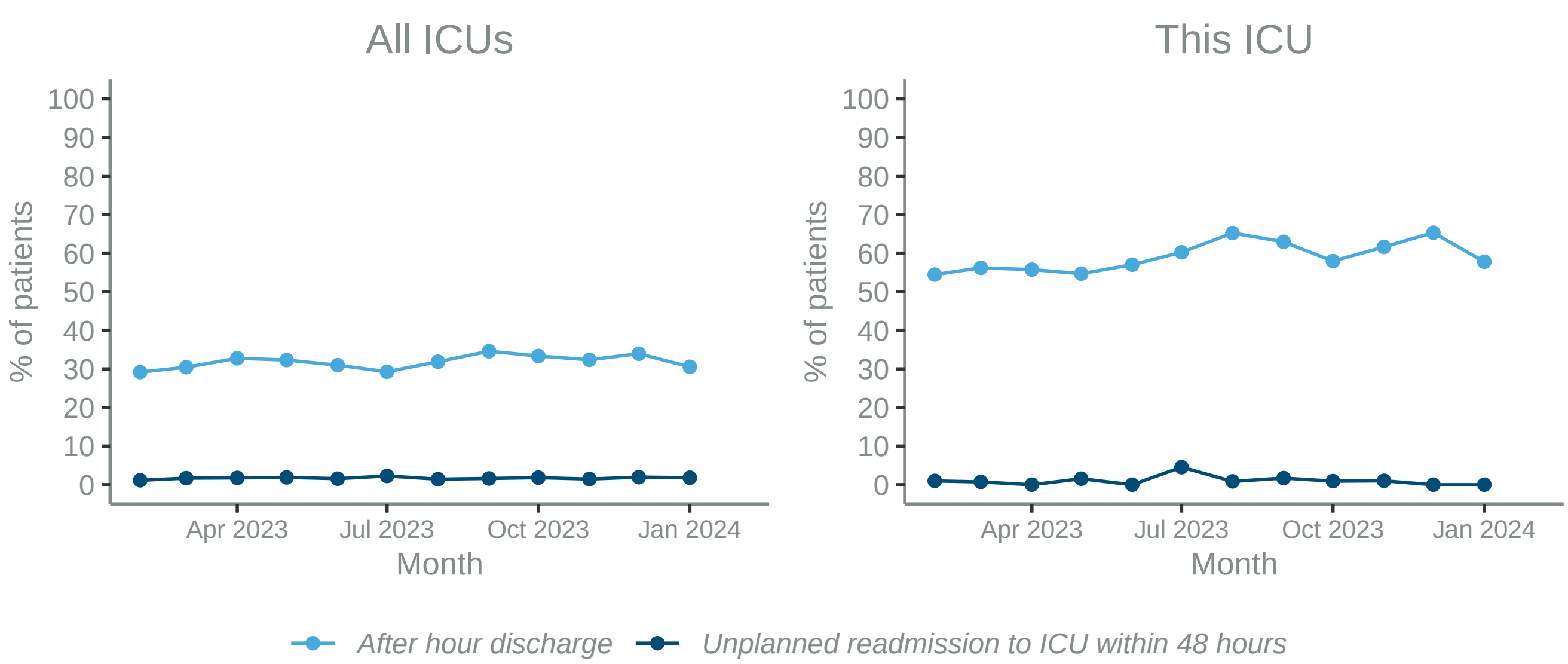

Figure 22: After hours discharge and unplanned readmission to ICU within 48 hours
After hours discharge defined as discharge between 7pm and 7am
