# Supplementary material for: Contextual determinants of implementation of stakeholder-selected quality indicators in Indian intensive care units: a mixed-methods evaluation within a national registry network
Source: Front Health Serv. 2026 Jul 27;6:1872641. doi: 10.3389/frhs.2026.1872641 (PMC13454447; doi:10.3389/frhs.2026.1872641)
Supplement: Supplementary file 3 [file Table1.docx]

**Date:**

**Time**

**Start:**

**End:**

**Site name** (code):

**Observer** (initials): VP

**Team members present** (note down roles, not names):

**Observation checklist**

| Note down which indicators are being mentioned while noting down the observations | |
| --- | --- |
| - Who was in the meeting? - Have new people joined/ has anyone been excluded? - Was the data for the indicators of care quality discussed? - How is the data feedback- Eg do the team use reports? / graphs/tables etc.? - What signs of engagement are there from the team? - Are there challenges with data collection? - How are these responded to? - Is there anything mentioned about the quality of the data collected, completeness, plausibility? - Are the definitions of the indicators discussed? - What influenced staff perceptions of the information presented to them? - Any individuals that had particularly positive or negative reactions to the information? - Do team members speak about the relative advantage/disadvantage for them as individuals of having the data? - Do they speak about relative advantage/disadvantages for the unit or organization? - Does the role / training/ hierarchy of the individuals affect their perspectives of the data? - How was the data related to clinical performance? - How was this information presented? Who presented it? - Was feedback relevant to individual staff, the team or the entire unit? - Was the clinical performance compared to anything (e.g. past performance, other units/hospitals/countries)? - What plans for service improvement are made? - Were the needs of the patients or perspective of the patients discussed regarding any of the quality indicators? |  |
