# Supplementary material for: Contextual determinants of implementation of stakeholder-selected quality indicators in Indian intensive care units: a mixed-methods evaluation within a national registry network
Source: Front Health Serv. 2026 Jul 27;6:1872641. doi: 10.3389/frhs.2026.1872641 (PMC13454447; doi:10.3389/frhs.2026.1872641)
Supplement: Supplementary file 4 [file Table2.docx]

# **Supplementary file 1: Table 1: The TIDieR (Template for Intervention Description and Replication) Checklist**

| Item number | Item | Description  Description |
| --- | --- | --- |
|  | **BRIEF NAME**  Provide the name or a phrase that describes the intervention. | Implementation of quality indicators using the IRIS online registry platformestablish data collection and the use of a bundle of six quality indicator for quality improvement in the ICUs of the participating sites (if sites want more or less, they can do so accordingly) |
| 1. | **WHY**  Describe any rationale, theory, or goal of the elements essential to the intervention.e.g. online appendix, URL). | To establish data collection and the use of a bundle of six quality indicator for quality improvement in the ICUs of the participating sites (if sites want more or less, they can do so accordingly)   - Indicator definition and data collection guidance - Implementation plan - Implementation checklist - MUSIQ 2 calculator   Observation sheets for audit and feedback. |
| 2. | **WHAT** | - Indicator aims table A |
| 3. | Procedures: Describe each of the procedures, activities, and/or processes used in the intervention, including any enabling or support activities.  For each category of intervention provider (e.g., psychologist, nursing assistant), describe their expertise, background and any specific training given. | Refer to table A Clinical Research Associates (CRAS) who have extensive hands-on experience working in ICUs to collect data and to interact with the various stakeholders in the ICU. CK from the backend team will be assisting the CRAs in technical platform related issues and AB and team will be available to help with engaging with the site leads. |
| 4. | **WHO PROVIDED?**  For each category of intervention provider (e.g., psychologist, nursing assistant), describe their expertise, background and any specific training given. | Training, follow up and engagement: These will be provided by the IRIS implementation team which consists of two clinical research associates (CRAS) who have extensive hands-on experience working in ICUs to collect data and to interact with the various stakeholders in the ICU.  CK from the backend team will be assisting the CRAs in technical platform related issues and AB and team will be available to help with engaging with the site leads.   - Registry platform to conduct the training and to conduct quality checks and data completeness checks   Establishing the use of the quality indicator data   - Video conferencing platform such as Zoom will be used for the orientation and the fortnightly meetings with the quality improvement team - Email: Site teams (site leads, DCs and Champions) will be contacted initially before the orientation using email, and once the team is formed, they will kept updated about any progress and all other meetings will be set up and meeting reports will be shared via email as well. - Registry platform will be used to show the quality improvement team the data completeness status and - Monthly reports (embedded with the registry): The reports will visualise the data collected in the registry. The numbers in the report will reflect how well the data is collected and the performance of the quality indicators in each ICU. |
| 5. | **HOW?**  Describe the modes of delivery (e.g. face-to-face or by some other mechanism, such as internet or telephone) of the intervention and whether it was provided individually or in a group. | Establishing the data collection of quality indicators seven hospitals or sites that have expressed their interest in implementing quality indicators through the IRIS platform (refer supplementary figure 1 for organogram)   - Phone and instant messaging: Regular communication and support will be provided through web-based messaging services (WhatsApp), and through phone calls as well. Sites are: (redacted as it is confidential) - Registry platform to conduct the training and to conduct quality checks and data completeness checks   Establishing the use of the quality indicator data   - Video conferencing platform such as Zoom will be used for the orientation and the monthly meetings with the quality improvement team - Email: Site teams (site leads, DCs and Champions) will be contacted initially before the orientation using email, and once the team is formed, they will kept updated about any progress and all other meetings will be set up and meeting reports will be shared via email as well. - Registry platform will be used to show the quality improvement team the data completeness status and - Monthly reports (embedded with the registry): The reports will visualise the data collected in the registry. The numbers in the report will reflect how well the data is collected and the performance of the quality indicators in each ICU.   Necessary infrastructure/features/resources   - Access to IRIS platform - Core and Daily Q assessment forms - Data collector to collect the data - Laptop/tab/phone to enter the data - A 3G internet connection or higher - Required permissions from the hospital to collect this data |
| 6. | **WHERE?**  Describe the type(s) of location(s) where the intervention occurred, including any necessary infrastructure or relevant features. Period of time including the number of sessions, their schedule, and their duration, intensity or dose. | The relevant locations are the seven hospitals or sites including 10 ICUs that met the following criteria: established daily data collection, presence of a dedicated data collector, and completeness of the data captured in the first 24 hours of admission is exceeding 95%. Other criteria considered were interest in quality indicator measurement, identification of an ICU champion (a doctor or an allied health professional) to liaise with the ICU, and an agreement to participate in monthly meetings to discuss the indicators. have expressed their interest in implementing quality indicators through the IRIS platform  The sites where the ICUs are located are: (redacted as it is confidential)  Necessary infrastructure/features/resources **meetings**: These meetings will take place once a month with a MDT staff members and will discuss the monthly report that will be generated. These meetings will last for 60 mins. Will be facilitated by the CRA and observed by the evaluation team.   - Access to IRIS platform - Core and Daily Q assessment forms - Data collector to collect the data - Laptop/tab/phone to enter the data - A 3G internet connection or higher : These visits will last for one week and will be conducted twice - the first within the first 12 weeks. The second round of visits will be planned, for 12-24 weeks |
| 7. | **WHEN and HOW MUCH?**  Describe the number of times the intervention was delivered and over what period of time including the number of sessions, their schedule, and their duration, intensity or dose. | **Site selection:** This will be done once at the start of the intervention. The CRA will look into the core completeness % of the sites and get in touch with them, determine their interest and then once selected will facilitate the selection of ICU Champions. This step will take 1-3 weeks |
| 9. | **MODIFICATIONS**  If the intervention was modified during the study, describe the changes (what, why, when, and how).strategies were used to maintain or improve fidelity, describe them. | Indicators collected: If the sites want to collect any additional quality indicators, then those will be added and a training for the data collectors for those additional variables will be done. In the same way, if any site wants to drop any of the indicators collected, that will be facilitated as well. First a discussion about why they wish to make the change will be arranged and then the necessary change will be done for the site.  If there are any issues faced by the sites due to the IRIS platform, then those issues will be taken to the development team and changes in the platform or strategies to sort the issues will be discussed and communicated to the site lead and data collector. |
| 10. | **HOW WELL** | Data completeness: The data completeness will be checked every week, and any missing data will be discussed with the data collectors. |
| 11.**r** | Actual: If intervention adherence or fidelity was assessed, describe the extent to which the intervention was delivered as planned.  **Description** | Data completeness was assessed at regular intervals  The monthly meetings were observed, and interviews were conducted to evaluate the barriers and facilitators which would explain the adherence and fidelity of the intervention. |

## **Table A: 3. WHAT: procedures**

| Time | Activity | Output |
| --- | --- | --- |
| Week 0 | Site selection   1. Identification of ICUs where Data collection electronic and daily established. 2. Identified DC present in the ICU 3. Completeness for ICU cohort core >95% 4. Interest in quality indicator measurement identified. 5. Champion (Dr and or AHP) identified. 6. Agreement for daily/ monthly meetings. | - Sites selected - ICU Champions identified - Meeting plans made |
| Weeks 1-2  Month 1-2  Month 1-2 | Site engagement and co-selection of indicators  DCs and the ICU champions select a bundle of 6 quality indicators from a list of indicators shared with them. Approval is sought from the Site leads of the participating sites and the national lead of IRIS for the indicators chosen.  Training of DCs and ICU Champions  After the indicators are chosen, data collectors and Champions are trained in the data entry of the quality indicator data using the IRIS registry platform. These meetings will be conducted virtually using zoom by the CRA and it will entail discussion of existing data entry mechanisms followed which will be recorded in the form of notes by the CRA. Then the training of how to collect data for the chosen indicators will be conducted.   1. Set up meeting 2. Ask if they have any questions about the project 3. If already collecting QI data, then ask them to take me through what they do and how they do it and how long they have been collecting the data 4. Introduce quality indicators and their definitions and explain through a few examples on how the data they collect is used to measure the quality indicators 5. Ask if they have any questions and clarify them 6. Take them through the test platform and explain the method to collect the data (one a daily basis, and how to enter each of the variables) - for sites already collecting focus on the things they need to improve on. 7. Once the training is completed and queries and doubts cleared, tell them that daily meetings will be held for establishing the data collection and fix a time for these daily meetings.   MUSIQ 2 assessment in each ICU.  This will be conducted by the evaluation team (VP) that will be working alongside the CRA. This will be an assessment that will determine the site’s readiness for quality improvement initiatives   1. Set up a meeting with the ICU team 2. Introduce the purpose of the assessment (to determine readiness for quality improvement) and explain that it will take 30-45 mins of their time and explain how the scoring system works 3. Ask them the questions and note down the score as they provide them for each question 4. Once the assessment is over thank them for their time and let them know that will get back to them with the score and what that means for their ICU.   Daily meetings with DCs and ICU Champions  These follow ups will be conducted via phone/WhatsApp/video conferencing depending on the needs of the DCs.  1. Ask about status of data collection for the previous day - completed? Yet to enter? Any issues with getting any of the data from the ICU sources to enter in the platform?  2. Note down/clarify any issues they may raise  3. Discuss any issues/queries observed by me during my check  4. If the data not entered - discuss why that is? Any issues faced? What is the frequency at which they enter the data? Strategies to minimise long gaps between data entries  After the meeting - if there is need to clarify any technical issues with Chamira and team - immediately raise it in an email  If there are any issues in accessing any data required then follow up via email with the site leads (the data collector, BK, AB, RH, ST looped in)  Make sure to close these loops within a weeks’ time or two weeks maximum  By the end of week 6 it is expected that the data collection will be established  During this process the CRA will work with VP to identify the solutions to the barriers identified and implement solutions with the involvement of the site leads, and national lead (BK)  Reports shared with Champions (Audit and feedback)  Reports regarding data completeness, and quality indicator data entered will be generated and shared with the Champions and discussions regarding the data quality, and challenges will be had between the CRA and the Champions. Also, discussions possible steps to improve both the data quality will be discussed.  The evaluation team (VP) will observe these Audit and Feedback sessions and take notes in an observation guide.  Co-design and revision of indicators  After these audit and feedback discussions the Champions and the CRA will investigate the indicators and will make any revisions in the chosen indicators if applicable. | The team is selected and oriented to the project, contact details are obtained and time and frequency of meetings are agreed upon  XX data collectors and XX ICU champions trained  The measurable output will be an assessment of the ICU (and its team's) readiness for quality improvement. This will include previous experience with QI, existing Audit and feedback activity, experience with engaging in change and improvement all of which may promote or inhibit the implementation process).  Data collection will be established. Barriers to data collection identified. Solutions identified and implemented  Solutions to challenges already implemented discussed.  Any other possible solutions to further challenges discussed and a plan to implement made  VP takes notes in the observation sheet which is developed based on CFIR framework  CRA and Champions identify any revisions and make the changes |
| Month 2 to 12 | Monthly reports and muti disciplinary team staff meetings  Monthly meetings will be facilitated in the selected sites discussion of monthly report will be a more in-depth discussion of the outputs of the data collected and how this data can be used to make changes in the ICU.   1. Show the report that will be shared with the team prior to the meeting and describe the data relating to their chosen indicators with them. In the first call say that this will be the baseline for their site, and we will be comparing any future reports with this one. 2. After each indicator pause and ask if they have question 3. Discuss about any gaps in data that are visible and plan to rectify it in the coming months 4. Discuss if there are any discrepancies regarding the report and the ICU data 5. Get their thoughts about the data, how it is useful for them, suggestions to make it more useful | Progress towards normalisation of use of indicator data in the ICU to monitor processes and outcomes,  Number of meetings convened over 12 weeks,  How clinical care improvements are prioritized, how many have been prioritized, and whether strategies have been put in place to remedy the issues that the indicators have identified. ,  Monthly report data used to track and compare ICUs performance of quality indicators every month for 3 months |
| Between months 8 and 12 | Implementation evaluation  Semi-structured interviews will be conducted via an online audio-conferencing platform with the site leads and data collectors and the ICU Champions that are implementing the quality indicators. The interview will be guided by the CFIR framework | Factors influencing the implementation identified. Recommendations for change to address the barriers suggested. |
| Month 12 | Repeat of the MUSIQ 2. | Measurable output will be change in score of MUSIQ 2 for each ICU |
