# Supplementary material for: Contextual determinants of implementation of stakeholder-selected quality indicators in Indian intensive care units: a mixed-methods evaluation within a national registry network
Source: Front Health Serv. 2026 Jul 27;6:1872641. doi: 10.3389/frhs.2026.1872641 (PMC13454447; doi:10.3389/frhs.2026.1872641)
Supplement: Supplementary file 5 [file Table3.docx]

Among the indicators that were implemented there were a few indicators that were derived from the core data in the first 24 hours of admission and day of discharge that are implemented irrespective of the sites selecting them. Table 3 shows the list of indicators that were selected (Y) and the indicators that were implemented (I) without being selected.

Table 1: Indicators selected by the sites

|  | Indicators chosen | **S1** | **S2** | **S3** | **S4** | **S5** | **S6** | **S7** | **Total implemented across sites** |
| --- | --- | --- | --- | --- | --- | --- | --- | --- | --- |
| **Foundation** | **Nursing staff to patient ratio*** | - | - | Y | - | Y | - | - | 0 |
| Foundation | Readmission within 48 hours of discharge | I | Y | Y | I | Y | Y | I | 7 |
| Process | VTE prophylaxis | Y | Y | Y | Y | Y | Y | Y | 7 |
| Process | Stress ulcer prophylaxis | Y | Y | Y | Y | Y | Y | Y | 7 |
| Process | Spontaneous Breathing Trial | Y | - | - | Y | Y | Y | - | 4 |
| Process | Spontaneous Awakening Trial | - | - | - | Y | - | - | - | 1 |
| Process | Duration of Mechanical ventilation | I | Y | Y | Y | Y | I | Y | 7 |
| Process | Duration of antimicrobial use | Y | Y | Y | Y | Y | - | Y | 6 |
| Process | Pressure injury | Y | Y | - | Y | - | Y | Y | 5 |
| Process | Daily Richmond ASS score | Y | Y | - | Y | - | - | Y | 4 |
| Process | Culture obtained within 24 hrs of antibiotic commencement | - | - | - | - | - | - | Y | 1 |
| Process | Accidental extubation | - | - | - | - | - | - | Y | 1 |
| Process | Daily position of bed and head elevation | - | - | - | - | - | - | Y | 1 |
| Process | CLABSI | - | - | - | - | - | - | Y | 1 |
| Process | Reintubation within 24 hrs of extubation | - | - | Y | - | - | Y | - | 2 |
| Process | Incidence of ICU-acquired drug-resistant organism of interest | - | Y | - | - | Y | - | - | 2 |
| **Quality impact** | **Post ICU quality of life*** | - | - | - | Y | - | - | - | 0 |
| Quality impact | No of patients being discharged from ICU due to lack of funds** | I | Y | Y | Y | I | I | I | 3 |
| Quality impact | Length of ICU stay | I | I | I | I | I | I | I | 7 |
| Quality impact | Risk adjusted ICU mortality (Standardized Mortality Ratio) | I | I | I | I | I | I | I | 7 |
|  | **Total selected in each site** | 6 | 9 | 8 | 10 | 8 | 6 | 10 |  |
|  | **Total implemented** | 11 | 11 | 9 | 13 | 9 | 10 | 14 |  |

*These indicators were chosen by some of the sites, but it was not made available for collection in the registry platform.

** Not reported in the monthly reports
